# The nutrient-responsive CDK Pho85 primes the Sch9 kinase for its activation by TORC1

**DOI:** 10.1371/journal.pgen.1010641

**Published:** 2023-02-15

**Authors:** Marie-Anne Deprez, Marco Caligaris, Joëlle Rosseels, Riko Hatakeyama, Ruben Ghillebert, Belém Sampaio-Marques, Kaivalya Mudholkar, Elja Eskes, Els Meert, Christian Ungermann, Paula Ludovico, Sabine Rospert, Claudio De Virgilio, Joris Winderickx

**Affiliations:** 1 Department of Biology, Functional Biology, KU Leuven, Heverlee, Belgium; 2 Department of Biology, University of Fribourg, Fribourg, Switzerland; 3 Institute of Medical Sciences, University of Aberdeen, Aberdeen, Scotland, United Kingdom; 4 Life and Health Sciences Research Institute (ICVS), School of Health Sciences, University of Minho, Braga, Portugal; 5 ICVS/3B’s—PT Government Associate Laboratory, Braga/Guimarães, Braga, Portugal; 6 Institute of Biochemistry and Molecular Biology, ZBMZ, Faculty of Medicine, University of Freiburg, Freiburg, Germany; 7 Department of Biology/Chemistry & Center of Cellular Nanoanalytics (CellNanOs), University of Osnabrück, Osnabrück, Germany; University College Dublin, IRELAND

## Abstract

Yeast cells maintain an intricate network of nutrient signaling pathways enabling them to integrate information on the availability of different nutrients and adjust their metabolism and growth accordingly. Cells that are no longer capable of integrating this information, or that are unable to make the necessary adaptations, will cease growth and eventually die. Here, we studied the molecular basis underlying the synthetic lethality caused by loss of the protein kinase Sch9, a key player in amino acid signaling and proximal effector of the conserved growth-regulatory TORC1 complex, when combined with either loss of the cyclin-dependent kinase (CDK) Pho85 or loss of its inhibitor Pho81, which both have pivotal roles in phosphate sensing and cell cycle regulation. We demonstrate that it is specifically the CDK-cyclin pair Pho85-Pho80 or the partially redundant CDK-cyclin pairs Pho85-Pcl6/Pcl7 that become essential for growth when Sch9 is absent. Interestingly, the respective three CDK-cyclin pairs regulate the activity and distribution of the phosphatidylinositol-3 phosphate 5-kinase Fab1 on endosomes and vacuoles, where it generates phosphatidylinositol-3,5 bisphosphate that serves to recruit both TORC1 and its substrate Sch9. In addition, Pho85-Pho80 directly phosphorylates Sch9 at Ser^726^, and to a lesser extent at Thr^723^, thereby priming Sch9 for its subsequent phosphorylation and activation by TORC1. The TORC1-Sch9 signaling branch therefore integrates Pho85-mediated information at different levels. In this context, we also discovered that loss of the transcription factor Pho4 rescued the synthetic lethality caused by loss of Pho85 and Sch9, indicating that both signaling pathways also converge on Pho4, which appears to be wired to a feedback loop involving the high-affinity phosphate transporter Pho84 that fine-tunes Sch9-mediated responses.

## Author summary

Cells possess different signaling pathways that sense and signal the availability of nutrients. Crosstalk between these pathways is essential to integrate the incoming signals and allow cells to make appropriate adaptations to sustain their metabolism and proliferation. In this study, we deciphered the crosstalk between two well-known nutrient-responsive pathways in yeast, namely the PHO pathway that signals the availability of phosphate via the cyclin-dependent protein kinase Pho85, and the TORC1 signaling pathway that communicates information on the availability of free amino acids via its downstream effector kinase Sch9. We show that Pho85 facilitates the TORC1-dependent activation of Sch9 through two different mechanisms. By interfering with the biosynthesis of the lipid phosphatidylinositol-3,5 bisphosphate, Pho85 controls the recruitment of Sch9 at the vacuolar membrane, thereby bringing this effector in close proximity to TORC1. In addition, Pho85 also directly phosphorylates Sch9, which primes the latter for its subsequent phosphorylation and activation by TORC1. Conversely, we provide evidence that the TORC1-Sch9 axis gives feedback to the PHO pathway by restraining the nuclear translocation of the transcription factor Pho4 that controls the expression of genes encoding proteins required to maintain phosphate homeostasis.

## Introduction

During the past decades, significant progress has been made in unravelling the dynamic and tightly regulated nutritional responses in yeast. These responses are controlled by a network of interconnected and conserved nutrient sensing routes that allow cells to adapt their metabolism in function of nutrient availability, thereby determining the growth potential and survival of cells.

A central role in the nutrient-responsive network of the yeast *Saccharomyces cerevisiae* is played by the protein kinase Sch9, which was suggested to combine the functions of the mammalian S6-kinase (S6K) [1] and protein kinase B (PKB)/Akt [2]. Sch9 controls several processes, including the regulation of transcription and translation [3–5], cellular stress responses [6–9], sphingolipid metabolism [10], pH homeostasis [11], and chronological as well as replicative lifespan [12,13]. Sch9 receives input from several upstream players. A first input is provided by the target of rapamycin complex 1 (TORC1), which signals nitrogen and amino acid availability and activates Sch9 by phosphorylation of at least 5 residues in the C-terminus [1]. Secondly, to gain full activity, Sch9 has to be phosphorylated in the activation loop by either one of the three phytosphingosine-dependent kinases, *i*.*e*. Pkh1, Pkh2, or Pkh3, the yeast orthologues of mammalian PDK1 [1,14,15]. Thirdly, the cellular energy sensor Snf1, the yeast AMPK orthologue, modulates Sch9 activity by phosphorylating residues that are distinct from those phosphorylated by TORC1 and Pkh1-3 [16–19]. Finally, the activity of Sch9 is also controlled by its recruitment to the vacuolar membrane where the kinase binds to phosphatidyl-inositol-3,5-bisphosphate (PI[3,5]P_2_), generated by the phosphatidylinositol-3-phosphate (PI3P) 5-kinase Fab1, the orthologue of the mammalian PIKfyve [20,21]. This recruitment is dependent on the N-terminal domain of Sch9 [22] and is essential for the TORC1-dependent activation of Sch9 [21,22]. Intriguingly, Fab1 is a substrate of TORC1, and its TORC1-dependent phosphorylation seems to control the distribution and shuttling of Fab1 between the vacuole and a subpopulation of prevacuolar endosomes, termed signaling endosomes [22–24]. At these signaling endosomes, Fab1 generates the main pool of PI[3,5]P_2_, which is subsequently delivered to the vacuole [22,24].

In a previous study, we reported on the genome-wide synthetic genetic array (SGA) analysis of *sch9Δ*. We noted that the combined deletion of *SCH9* with either the cyclin-dependent kinase (CDK) inhibitor *PHO81* or the CDK *PHO85* resulted in a synthetic lethal phenotype [11]. Pho81 and Pho85 are key players in the phosphate-responsive signaling pathway, known as the PHO pathway, that regulates the expression of genes required to maintain proper phosphate homeostasis. In this pathway, the CDK inhibitor (CKI) Pho81 becomes active when phosphate is limiting and inhibits the activity of the CDK–cyclin pair Pho85–Pho80, thereby enabling the transcription factor Pho4 to localize in the nucleus and induce the expression of genes required for the foraging, import, and storage of extracellular phosphate and the recycling of intracellular phosphate [25–27]. Notably, Pho81 also controls the activity of the Pho85-Pcl7 CDK-cyclin pair, which is suggested to be involved in phosphate sensing as well given its ability to phosphorylate Pho4 *in vitro* [28,29]. Our observation of a synthetic lethality between *pho81Δ* or *pho85Δ* and *sch9Δ* indicates that both hyperactivation and disruption of Pho85 is detrimental for cell survival in the absence of Sch9 activity and is in line with multiple observations linking phosphate sensing to other nutrient-responsive pathways [30]. Hence, the activities of Pho85 and the TORC1-Sch9 axis are required to be critically balanced and coordinated.

Pho85 is involved in the regulation of many different aspects of cell cycle control and environmental signaling [31–37]. Its deletion results in numerous defects, which besides altered phosphate metabolism, also includes slow growth, inability to grow on non-fermentable carbon sources, cell cycle defects, abnormal cell morphology and cell wall integrity, enhanced sensitivity to several types of stress, altered lipid metabolism, compromised reserve carbohydrate accumulation, as well as reduced autophagy and longevity [31,38–44]. Each of these defects can be linked to the function of specific Pho85-cyclins directing the Pho85 kinase to specific substrates [45–48]. The cyclins have been divided into two groups based on their sequence similarity: the Pho80-like subfamily, which besides Pho80 and Pcl7 also includes Pcl6, Pcl8 and Pcl10, and the Pcl1,2-like subfamily, which contains Pcl1, Pcl2, Pcl5, Pcl9, and Clg1 [46]. The Pho80-like cyclins and Pcl5 are involved in the regulation of metabolism in response to environmental changes, while the Pcl1,2-like cyclins are mainly connected to cell cycle control and morphogenesis [29,46,49]. Interestingly, the Pho85-Pho80 CDK-cyclin complex can phosphorylate and boost the activity of Fab1 under hyperosmotic stress conditions [50], suggesting that Fab1 could act as a possible point of convergence with the TORC1 signaling cascade.

In this study, we explored the synthetic lethality caused by the combined deletion of *SCH9* and either *PHO85* or *PHO81*. We demonstrate that these synthetic lethalities are due to conflicting signals in the crosstalk between Pho85, TORC1, and Sch9. We provide evidence that the CDK-cyclin pair Pho85-Pho80 directly phosphorylates Sch9 to prime this kinase for subsequent phosphorylation by TORC1. In addition, we confirm that Pho85-Pho80 affects Fab1 activity and provide evidence that also Pho85-Pcl6 and Pho85-Pcl7 are likely involved in the regulation of the cellular PI[3,5]P_2_ levels as judged from the vacuolar recruitment of Sch9. Finally, we show that the transcription factor Pho4 is not only a downstream target of Pho85 signaling but of TORC1-Sch9 signaling as well.

## Results

### Crosstalk between Pho85 and the TORC1-Sch9 axis involves the cyclins Pho80, Pcl6, and Pcl7

**Fig 1:**
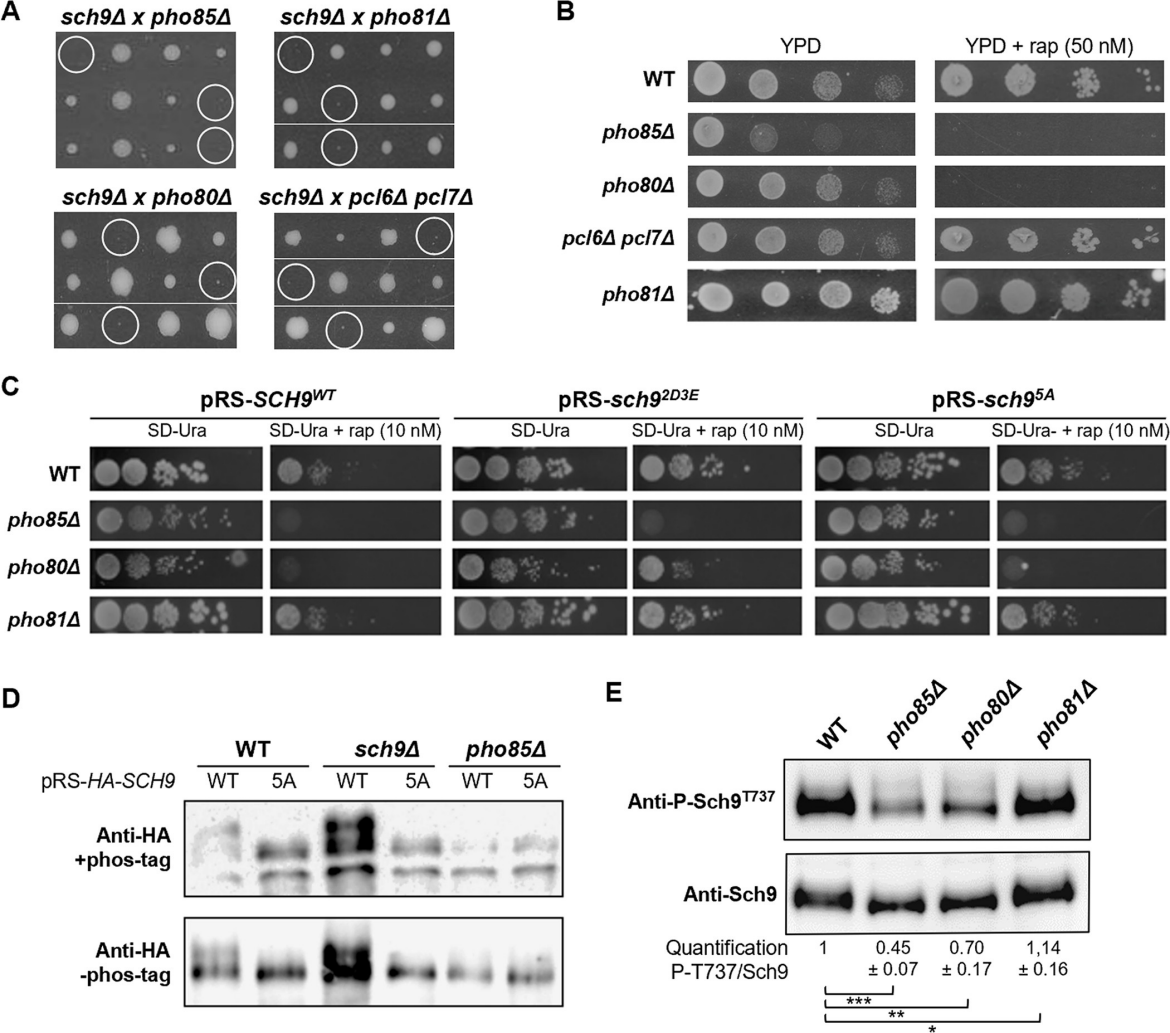
Genetic interaction between the Pho85 and Sch9 signaling pathways. (A) Tetrad analysis on YPD plates of a cross between the *sch9Δ* mutant and mutant strains lacking either Pho81, Pho85, Pho80, or the combination of Pcl6 and Pcl7. The spores that combine the deletions of the parental mutant strains and define the synthetic lethal or synthetic sick phenotype are indicated by the white circles. Pictures were taken 3 to 5 days after dissection. See S1 Fig for the complete genotypic analysis of all cyclin mutants. (B) Rapamycin sensitivity analysis of the WT (BY4741), *pho85Δ*, *pho80Δ*, *pcl6Δ pcl7Δ*, and *pho81Δ* strains. The strains were grown to exponential growth phase on YPD medium, diluted to an OD_600nm_ of 0.1 and serial 10-fold dilutions were then spotted on YPD plates without or with 50 nM rapamycin (rap) and grown at 30°C. See S2 Fig for the rapamycin sensitivity test of all cyclin mutants. (C) Rapamycin sensitivity analysis of the WT, *pho85Δ*, *pho80Δ*, and *pho81Δ* strains transformed with centromere plasmids allowing for expression of either Sch9^WT^, Sch9^2D3E^, or Sch9^5A^, and assessed by growth on SD medium lacking uracil (SD-ura) without or with 10 nM rapamycin. (D) Phos-tag immunoblot analysis to assess Sch9 phosphorylation levels in protein extracts obtained from exponentially growing WT, *sch9Δ*, and *pho85Δ* cells expressing either HA-Sch9^WT^ or HA-Sch9^5A^. The protein extracts were resolved on phos-tag gels and subsequently analyzed via immunoblotting with anti-HA antibodies. (E) Immunoblot analysis of protein extracts obtained from exponentially growing WT, *pho85Δ*, *pho80Δ*, and *pho81Δ* cells using the anti-P-Sch9^T737^ and anti-Sch9 antibodies. The quantifications show the ratio of phosphorylated to total Sch9 as normalized to the ratio obtained for the WT cells. A two-tailed student’s T test was used to calculate significances (*, P < 0.1; **, P < 0.01; ***, P < 0.001).

To confirm the previously reported genetic interactions between Sch9, Pho81, and Pho85, and to identify which Pho85 cyclins contribute to the observed effects, we crossed the *sch9Δ* strain with isogenic strains lacking *PHO81*, *PHO85*, or either one of the known cyclins, and performed a systematic tetrad analysis. As shown in Fig 1A, the synthetic lethal phenotype of the *sch9Δ pho81Δ* and *sch9Δ pho85Δ* strains was mimicked by the combined deletion of *SCH9* and *PHO80*. The *sch9Δ pho80Δ* spores were still able to germinate but showed a very severe synthetic growth defect. For all other cyclins, the combined deletion with *SCH9* yielded viable spores that did not exhibit significant growth differences in comparison to the *sch9Δ* strain (S1 Fig). However, since the cyclins Pcl1 and Pcl2, Pcl6 and Pcl7, or Pcl8 and Pcl10 have partially redundant functions [28,47,51], we also tested their combined deletions. While the combined deletion of *PCL1* and *PCL2*, or of *PCL8* and *PCL10* in the *sch9Δ* background did not further exacerbate the slow growth phenotype of the *sch9Δ* mutant, a pronounced synthetic growth defect was noticed in case of the combined *PLC6* and *PLC7* deletion (Fig 1A, S1 Fig). Hence, we can conclude that loss of the cyclin functions of Pho80 and a combination of Pcl6 and Pcl7 contribute to the synthetic lethal phenotype of the *sch9Δ pho85Δ* strain.

Because Sch9 activity is dependent on its phosphorylation by TORC1 [1], the growth of the *pho81Δ*, *pho85Δ*, and cyclin deletion strains was also monitored in the presence of sub-lethal levels of rapamycin, a specific inhibitor of TORC1. As shown in Fig 1B, both the *pho85Δ* and *pho80Δ* cells were unable to grow on rich medium supplemented with 50 nM rapamycin, which is consistent with previously made observations [40,43]. In contrast, wild-type cells (BY4741; WT) and the other strains carrying either single or double cyclin deletions or a *PHO81* deletion did not display rapamycin-sensitive growth (S2A and S2B Fig). This is an intriguing observation, because it suggests that under these sub-lethal conditions there is still sufficient Sch9 activity to maintain growth of the *pho81Δ* and *pcl6Δ pcl7Δ* strains.

Next, we investigated whether the rapamycin-induced growth defect of the *pho85Δ* and *pho80Δ* strains could be restored by transforming the strains with a centromere plasmid expressing the constitutive active TORC1 phosphomimetic Sch9^2D3E^ mutant [1]. As a control, strains were also transformed with plasmids expressing wild-type Sch9^WT^ or the Sch9^5A^ mutant that cannot be activated by TORC1. As shown in Fig 1C, neither the expression of Sch9^WT^ nor Sch9^5A^ could restore the rapamycin-induced growth defect of the strains, while overexpression of Sch9^2D3E^ clearly improved growth in the presence of rapamycin in case of the *pho80Δ* strain, but not in case of the *pho85Δ* strain. For comparison, we also included the WT and the *pho81Δ* strain in this experiment and, as expected, their growth on rapamycin-containing medium was slightly improved by the expression of the Sch9^2D3E^ allele (Fig 1C). Thus, the observation that Sch9^2D3E^ expression rescues the rapamycin sensitivity of the *pho80Δ* strain suggests that the Pho85-Pho80 CDK-cyclin pair may be specifically required for TORC1-mediated phosphorylation and activation of Sch9. To address this possibility, we performed a Phos-tag mobility shift analysis using protein extracts of WT, *sch9Δ*, and *pho85Δ* cells expressing HA-tagged constructs of either Sch9^WT^ or the Sch9^5A^ mutant that served as control (Fig 1D). This clearly demonstrated that the phosphorylation of Sch9 was compromised in the *pho85Δ* strain because only in this strain the slowly migrating band corresponding to fully phosphorylated Sch9^WT^ was absent, resulting in a similar mobility pattern for Sch9^WT^ as that seen for Sch9^5A^. Consistently, immunodetection of native Sch9 and of its phosphorylation state at the TORC1 residue Thr^737^ (using anti-Sch9 and anti-phospho-Sch9^T737^ antibodies, respectively), demonstrated that Sch9 phosphorylation was significantly reduced in the *pho85Δ* and the *pho80Δ* strain, while being enhanced in the *pho81Δ* (Figs 1E and S2C).

### Pho85-Pho80 is required for vacuolar recruitment of Fab1 and Sch9

**Fig 2:**
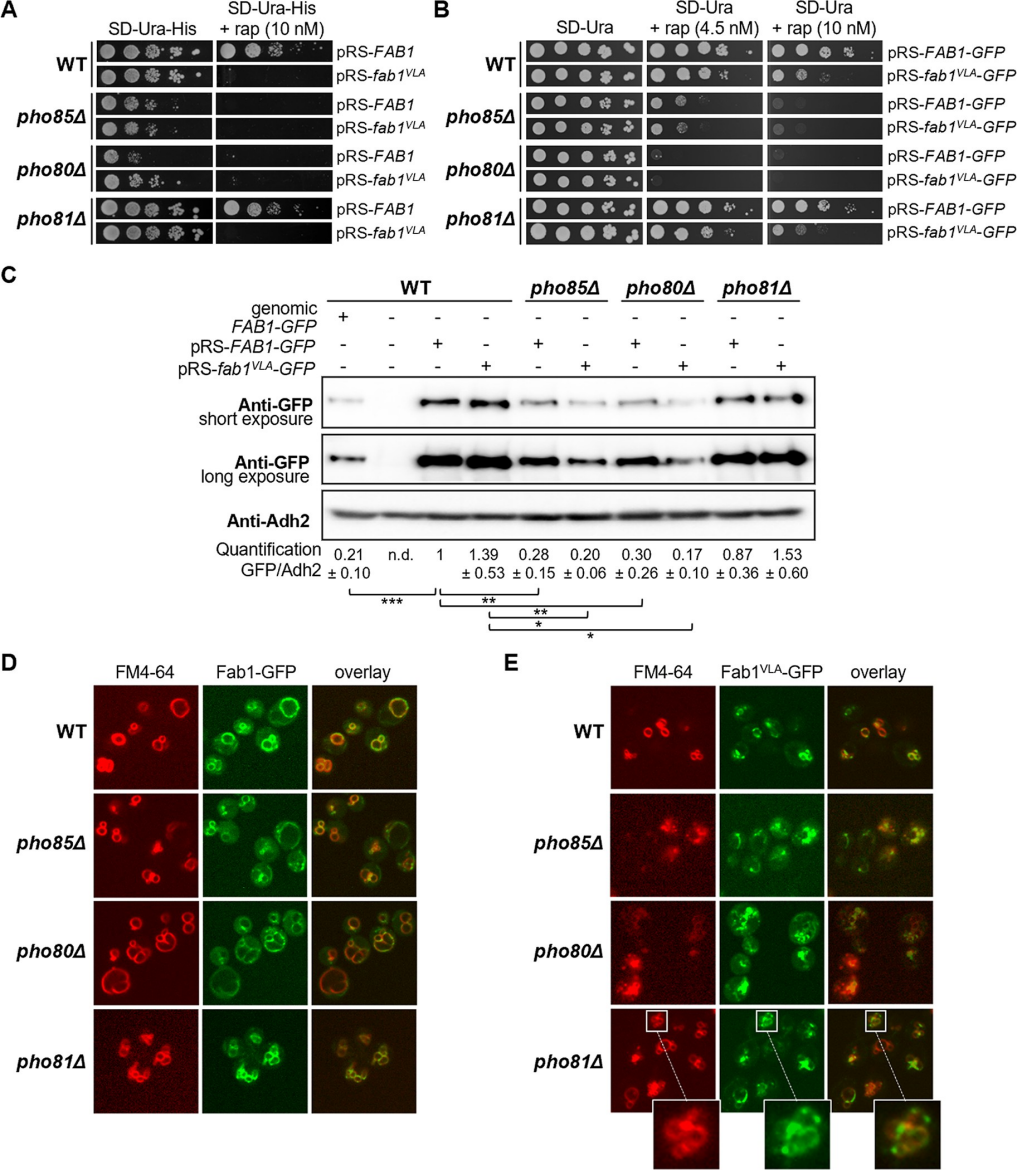
Pho85-Pho80 is involved in the recruitment of Fab1 to the vacuolar membrane. (A) Rapamycin sensitivity analysis of the WT, *pho85Δ*, *pho80Δ*, and *pho81Δ* strains expressing Sch9^WT^ and Fab1 alleles from centromere plasmids. Growth was assessed on selective synthetic medium without or with 10 nM rapamycin (rap). (B) Rapamycin sensitivity analysis in the presence of 4.5 nM or 10 nM rapamycin of the WT, *pho85Δ*, *pho80Δ*, and *pho81Δ* strains expressing either the GFP-tagged wild-type Fab1 or the GFP-tagged Fab1^VLA^ allele from a centromere plasmid as indicated. (C) Immunoblot analysis of the WT, *pho85Δ*, *pho80Δ*, and *pho81Δ* strains to compare the expression levels of the Fab1-GFP and Fab1^VLA^-GFP fusions when introduced on centromere plasmids with the expression level of a genomically tagged Fab1-GFP present in the WT strain. Expression levels were calculated based on the ratios obtained for GFP and the loading control Adh2. A two-tailed student’s T test was used to calculate significances (*, P < 0.1; **, P < 0.01; ***, P < 0.001). (D) Microscopic analysis of Fab1-GFP and Fab1^VLA^-GFP localization in the WT, *pho85Δ*, *pho80Δ*, and *pho81Δ* strains. The strains were grown to mid-log phase on selective synthetic medium. The lipophilic dye FM4-64 was used to visualize the vacuolar membrane. The indents in the pictures of Fab1^VLA^-GFP expressing *pho81Δ* cells are magnifications to clarify that this hyperactive Fab1 mutant largely fails to stain vacuolar membranes and is mainly localized in foci close to, or at the vacuole.

Previous studies indicated that Sch9 is recruited to the vacuolar membrane during fermentative growth where it binds PI[3,5]P_2_ via its N-terminal domain and then becomes phosphorylated by TORC1 [1,21,22,52]. PI[3,5]P_2_ is generated from PI3P by the PIKfyve-like kinase Fab1, whose activity is tightly regulated by intramolecular inhibitory interactions and by different regulatory proteins that form a complex with Fab1 [53–57]. Both TORC1 and Pho85-Pho80 impact on the Fab1 activity [22, 50]. Previous research demonstrated signaling endosomes to be the main site for PI[3,5]P_2_ production [22]. These signaling endosomes contain the EGO complex and TORC1, which phosphorylates Fab1 in the N-terminal half close to the FYVE (Fab1, YOTB, Vac1 and EEA1) domain, thereby enhancing the PI3P binding of Fab1 and promoting the PI[3,5]P_2_ generation. According to the current working model, PI[3,5]P_2_ is delivered to the vacuolar membrane upon fusion of the signaling endosome with the vacuole, and the EGO complex, TORC1, and Fab1 become dispersed over the vacuolar membrane. Fab1 is then recycled back to the signaling endosome in order to restart PI[3,5]P_2_ production [22]. Pho85-Pho80 is known to boost the activity of Fab1 by phosphorylation in the C-terminal region close to the catalytic kinase domain, thereby enhancing PI[3,5]P_2_ production upon stress [50]. In addition, Pho85-Pho80 also phosphorylates Vac7, a positive regulator of Fab1 [50]. Thus, to address the possibility that Pho85-Pho80 affects the TORC1-dependent phosphorylation of Sch9 indirectly through the regulation of Fab1, we wondered whether overactivation of Fab1 would restore the rapamycin-induced growth defect of the *pho85Δ* and *pho80Δ* strains. To make the comparison with the aforementioned growth assay (Fig 1C), we again transformed the WT, *pho85Δ*, *pho80Δ*, and *pho81Δ* strains with the centromere plasmid encoding Sch9^WT^ but this time together with a centromere plasmid providing either additional copies of wild-type Fab1 or the hyperactive Fab1^VLA^ mutant that was reported to yield more than 10-fold increased basal PI[3,5]P_2_ levels [55]. As shown in Fig 2A, neither wild-type Fab1, nor the Fab1^VLA^ mutant allowed the *pho85Δ* or the *pho80Δ* strains to grow on medium supplemented with 10 nM rapamycin. In fact, we noticed that the Fab1^VLA^ mutant even caused rapamycin sensitivity in the WT and *pho81Δ* strain. Both observations incited us to monitor the expression of the Fab1 and Fab1^VLA^ proteins in more detail. Since currently no Fab1 antibody is available, we transformed the different strains with centromere plasmids allowing the expression of both Fab1 proteins as C-terminally tagged GFP fusion under control of the *FAB1* promotor. When assayed for growth in the presence of 10 nM rapamycin, similar results were obtained as before, *i*.*e*. no growth in case of the *pho85Δ* and *pho80Δ* strains and enhanced sensitivity for the WT and *pho81Δ* strain when expressing the Fab1^VLA^-GFP fusion (Fig 2B). We further noted that the *pho85Δ* strain was slightly less sensitive to rapamycin than the *pho80Δ* strain when grown on lower levels (*i*.*e*. 4.5 nM) of rapamycin (Fig 2B). Next, we used an anti-GFP antibody to estimate the expression levels of the Fab1-GFP and Fab1^VLA^-GFP fusions in the different strains using Adh2 as loading control. When compared to genomically expressed Fab1-GFP levels in WT cells, centromere plasmid-expressed Fab1-GFP levels appeared to be roughly 5-fold higher in WT, 4-fold higher in *pho81Δ*, and 1.5-fold higher in *pho85Δ* and *pho80Δ* cells (Fig 2C). In WT and *pho81Δ* cells, the plasmid-expressed Fab1^VLA^-GFP levels were even slightly higher than the ones observed for plasmid-expressed Fab1-GFP, but they were somewhat lower than the respective plasmid-expressed Fab1-GFP levels in both *pho85Δ* and *pho80Δ* cells (Fig 2C). Thus, even though *pho85Δ* and *pho80Δ* cells exhibit plasmid-expressed Fab1-/Fab1^VLA^-GFP levels that are in a comparable range to the ones of genomically expressed Fab1-GFP in WT cells, they appear, unlike WT and *pho81Δ* cells, unable to support (plasmid-driven) expression of much higher Fab1-/Fab1^VLA^-GFP levels. We then monitored the intracellular localization of the Fab1-GFP and Fab1^VLA^-GFP fusions in the different WT and mutant strains (Fig 2D and 2E). In line with the measured expression levels, Fab1-GFP was present on vacuolar membranes in all strains, but the staining was on average less intense in *pho85Δ* and *pho80Δ* cells as compared to WT and *pho81Δ* cells. Furthermore, in *pho85Δ* cells and *pho80Δ* cells, Fab1-GFP mainly localized at small vacuoles as well as in foci close to, or at the vacuolar membrane. These foci probably correspond to the previously reported signaling endosomes [22,24]. If confirmed, we deem it reasonable to assume that the lack of Pho85 or Pho80 impedes the fusion of these perivacuolar endosomes and as such the distribution or stabilization of Fab1 at vacuolar membranes. As expected, and consistent with previous observations [50,55], cells expressing the hyperactive Fab1^VLA^-GFP version displayed small and tiny, almost vesicle-like, vacuoles. Interestingly, Fab1^VLA^-GFP was to a large extent absent from membranes of the discernible vacuoles and almost exclusively present in foci, and this independently of the presence or absence of Pho85, Pho80, or Pho81. This defect is unlikely caused by the GFP tag because wild-type Fab1-GFP properly localized to vacuolar membranes and, because both GFP-tagged and untagged Fab1^VLA^ caused rapamycin-sensitivity to a similar extent in all strains studied. However, we noted that the mutations in the Fab1^VLA^ allele (E1822V, F1833L, T2250A; [55]) are located within the reported cluster of potential Pho85-Pho80 target residues (T1569, T1583, T1594, T1691, S1924, T1953, T1963, and S2166; [50]). It is therefore possible that the mutations in this Fab1 variant mimic the phosphorylation by Pho85-Pho80 and that this prevents the stabilization of Fab1 at the vacuolar membrane resulting in a continuous recycling back to the perivacuolar signaling endosome to generate more PI[3,5]P_2_, a model that remains to be addressed in future studies. If true, then the phosphorylation of Fab1 by Pho85-Pho80 not only stimulates the fusion of signaling endosomes to the vacuole but also promotes the localization of Fab1 at perivacuolar signaling endosomes. Such a model would also elegantly explain why cells with hyperactive Pho85 (e.g., due to the lack of the CKI Pho81) mostly display smaller and fragmented vacuoles (Fig 2D and 2E). Thus, our combined data suggest that the equilibrium of endosomal and vacuolar Fab1 is critically controlled by both TORC1, as previously reported [22], and Pho85-Pho80.

Finally, the enhanced rapamycin sensitivity observed for the WT strain when expressing Fab1^VLA^ led us to monitor the Sch9^T737^ phosphorylation levels. We found these to be slightly lower in cells expressing Fab1^VLA^ as compared to cells with the empty vector control or cells expressing Fab1, suggesting that hyperactivation of Fab1 is associated with reduced TORC1 activity (S3A Fig). A similar but more pronounced effect was previously observed for cells expressing the Fab1^6D^ mutant that also displays enhanced Fab1 activity [22].

**Fig 3:**
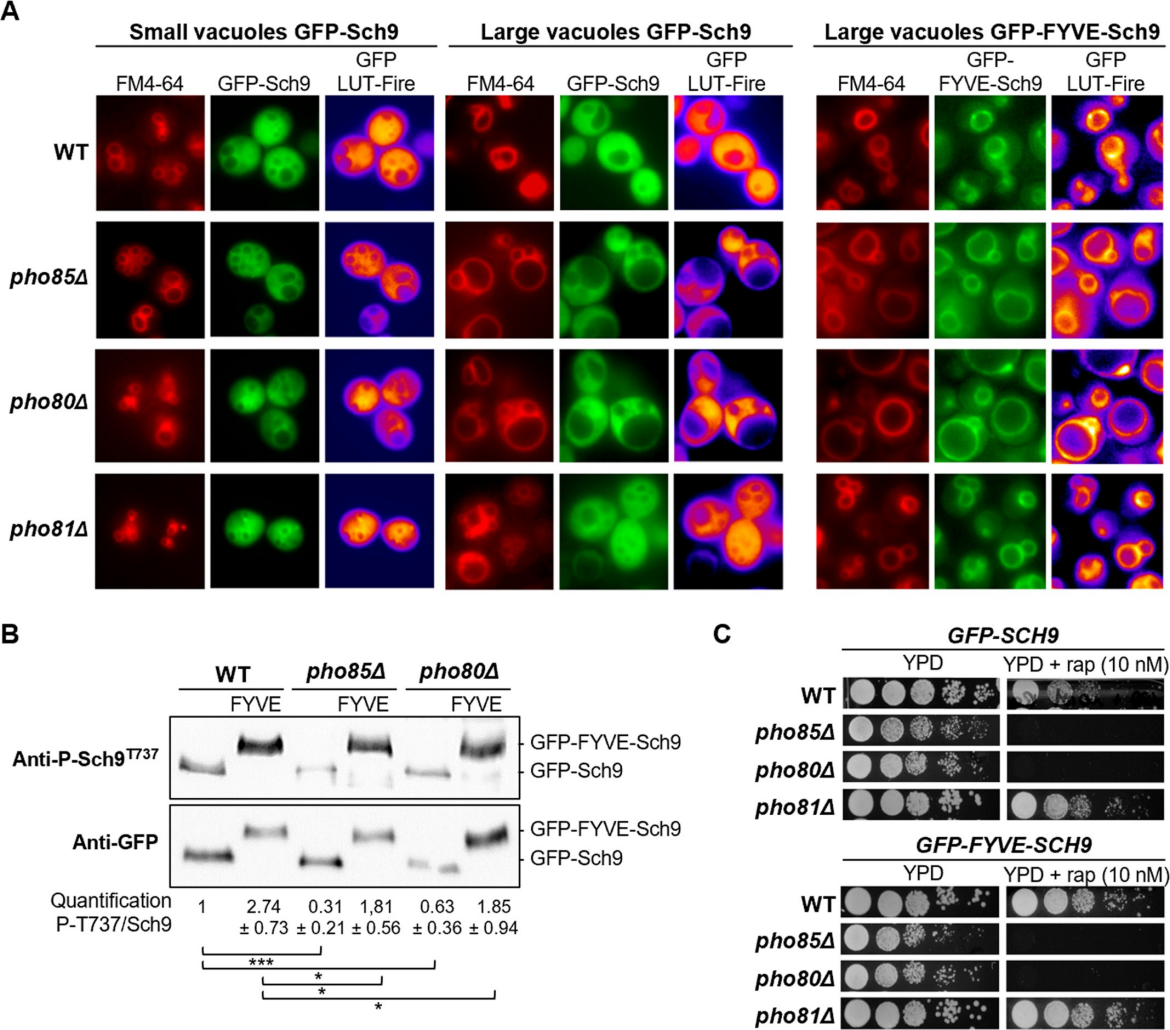
Pho85-Pho80 is required for the vacuolar recruitment of Sch9. (A) Microscopic analysis of Sch9 localization in the WT, *pho85Δ*, *pho80Δ*, and *pho81Δ* strains expressing genomically tagged GFP-Sch9 or GFP-FYVE-Sch9 fusion proteins. Strains were grown to mid-log phase on complete synthetic medium. The lipophilic dye FM4-64 was used to visualize the vacuolar membrane and a LUT Fire was applied using ImageJ to show the levels of the GFP signal. (B) Immunoblot analysis to compare the Sch9 phosphorylation levels in WT, *pho85Δ*, and *pho80Δ* cells expressing either GFP-Sch9 or GFP-FYVE-Sch9 when grown to mid-log phase on complete synthetic medium. The Sch9-Thr^737^ phosphorylation levels were quantified based on the ratio of the signals obtained with the anti-P-Sch9^T737^ and anti-Sch9 antibodies and normalized to WT cells. A two-tailed student’s T test was used to calculate significances (*, P < 0.1; **, P < 0.01; ***, P < 0.001). (C) Rapamycin sensitivity analysis of the WT (BY4741), *pho85Δ*, *pho80Δ*, and *pho81Δ* strains expressing genomically tagged GFP-Sch9 or GFP-FYVE-Sch9 as assessed by growth on YPD plates without or with 10 nM rapamycin (rap).

In line with our data on Fab1-GFP localization and a model in which Pho85-Pho80 is an upstream activator of Fab1 that boosts the PI[3,5]P_2_ content of the vacuolar membrane, thereby determining vacuolar size and morphology [50,58,59], we noticed that in comparison to the WT or *pho81Δ* strains, the *pho85Δ* strain and especially the *pho80Δ* strain had many cells with enlarged vacuoles. In contrast to cells displaying small and fragmented vacuoles, the cells with these enlarged vacuoles appeared to be hampered for the vacuolar recruitment of a genomically tagged GFP-Sch9^WT^ (Figs 3A and S3B). Likewise, we also found lower GFP-Sch9 levels at the membranes of vacuoles when these were isolated from cells lacking Pho85 (S3C and S3D Fig). Given that Sch9 normally needs to bind PI[3,5]P_2_ at the vacuolar membrane, we wondered whether forced anchoring of Sch9 to the vacuolar membrane would be sufficient to correct the reduced Sch9 phosphorylation by TORC1 as seen in the *pho85Δ* and *pho80Δ* strains and thereby resolve their rapamycin sensitivity. To address this, a genomically tagged GFP-FYVE-Sch9^WT^ was introduced in the WT, *pho85Δ*, *pho80Δ*, and *pho81Δ* strains. As described previously, fusing the FYVE domain from mammalian EEA1 to the N-terminus of Sch9, artificially tethers the kinase to PI3P in the vacuolar membranes of yeast cells [60]. As such, we indeed observed a strong vacuolar enrichment of GFP-FYVE-Sch9^WT^, even in *pho85Δ* and *pho80Δ* cells with enlarged vacuoles, which now displayed fluorescence over the entire vacuolar membrane (Figs 3A and S3B). The artificial tethering came along with dramatically enhanced phosphorylation levels of GFP-FYVE-Sch9^WT^ at the Thr^737^ residue in all the strains, but still, this was significantly lower in the *pho80Δ* and *pho85Δ* strains as compared to the WT strain (Fig 3B). Furthermore, despite the enhanced phosphorylation, GFP-FYVE-Sch9^WT^ did not alleviate the rapamycin sensitivity of the *pho85Δ* and *pho80Δ* strain (Fig 3C), suggesting that the Pho85-Pho80 CDK-cyclin pair could also (directly or indirectly) target Sch9, besides Fab1. This possibility is further supported by our initial observation that expression of the TORC1 phosphomimetic Sch9^2D3E^ mutant rescues the rapamycin sensitivity of the *pho80Δ* strain (Fig 1C).

### Pho85-Pho80 primes Sch9 for phosphorylation by TORC1 at the vacuolar membrane

**Fig 4:**
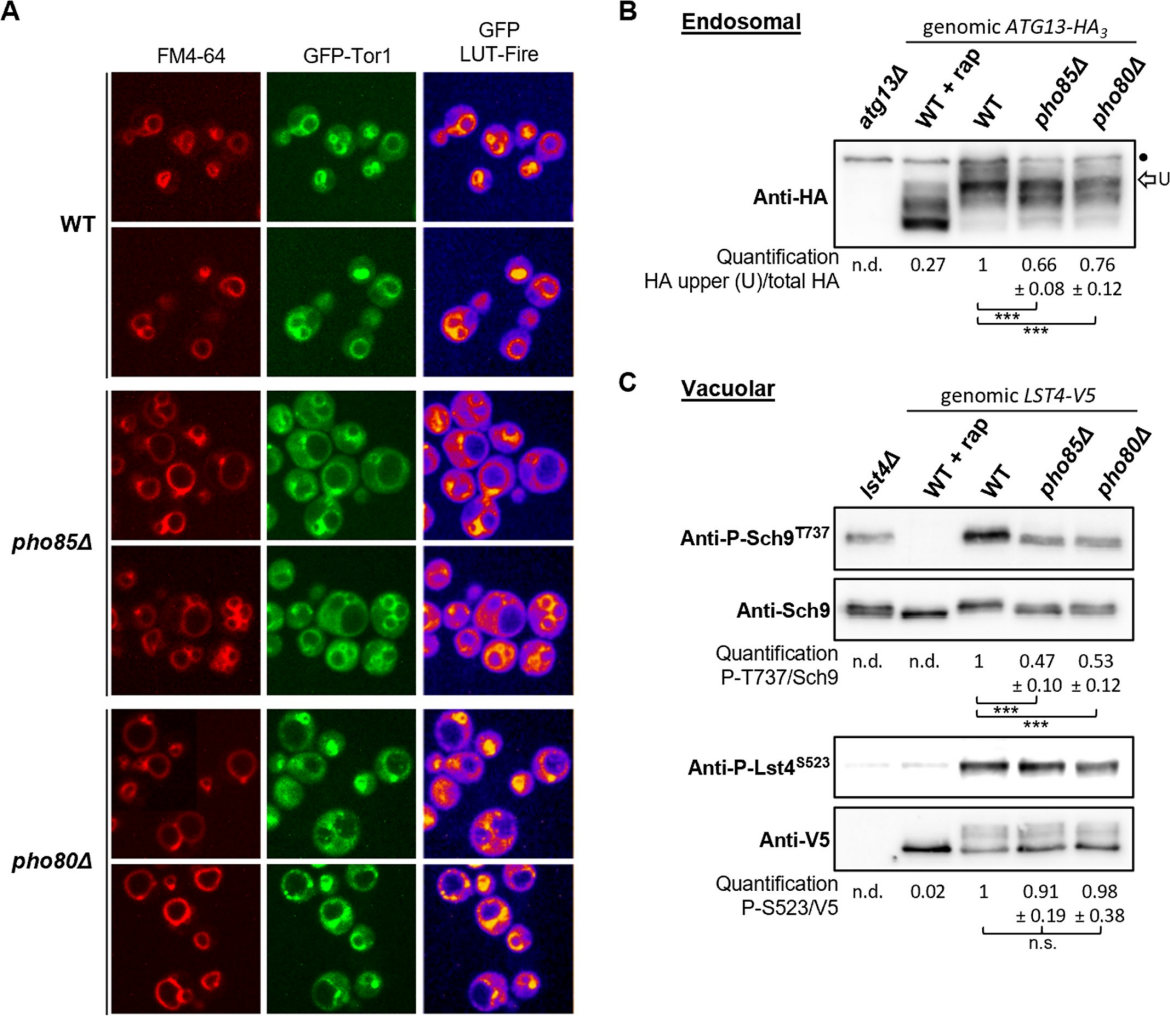
Tor1 localization and TORC1 activity in WT, *pho85Δ*, and *pho80Δ* strains. (A) Microscopic analysis of Tor1 localization in the WT, *pho85Δ*, and *pho80Δ* strains expressing a genomically tagged GFP-Tor1 fusion. Strains were grown to mid-log phase on complete synthetic medium. The lipophilic dye FM4-64 was used to visualize the vacuolar membrane and a LUT Fire was applied using ImageJ to visualize the levels of the GFP signal. (B, C) Immunoblot analyses to compare the Atg13 (B), or Sch9 and Lst4 (C) phosphorylation levels in WT, *pho85Δ*, and *pho80Δ* cells expressing either a genomically introduced Atg13-HA_3_ or Lst4-V5 fusion when grown to mid-log phase on complete synthetic medium. The dot in the Atg13-HA_3_ blots indicate a non-specific cross-reacting band, the arrow ‘U’ points to the bands corresponding to the most phosphorylated Atg13-HA_3_ isoforms that was quantified and used to calculate the ratio relative to the total HA signal. For Sch9 and Lst4, the phosphorylation levels were quantified based on the ratio of the signals obtained with the anti-P-Sch9^T737^ or anti-P-Lst4^S523^, and the anti-Sch9 or anti-V5 antibodies, respectively, and normalized to the one in WT cells. As indicated, the *atg13Δ* strain, the *lst4Δ* strain, and the WT strain expressing either genomic Atg13-HA_3_ or Lst4-V5, but treated with 200 nM rapamycin (rap) for 30 min, served as controls. A two-tailed student’s T test was used to calculate significances (*, P < 0.1; **, P < 0.01; ***, P < 0.001, n.s.: not significant).

To further support that Sch9 is a substrate for Pho85-Pho80, we first wanted to rule out the possibility that the observed decreased phosphorylation of Sch9 in the *pho85Δ* and *pho80Δ* strains would simply be due to a reduced TORC1 activity at the vacuolar membrane. As mentioned above, the EGO complex and TORC1 are mainly present in different pools. In prevacuolar endosomes, both complexes have been described to decorate signaling endosomes, and the HOPS-mediated fusion of these endosomes with the vacuole determines the pool of the latter [22–24]. We first monitored the intracellular localization of a genomically tagged GFP-Tor1 in WT, *pho85Δ*, and *pho80Δ* strains. In WT cells, the GFP-Tor1 fusion nicely stained the membranes of all vacuoles. In the *pho85Δ* and the *pho80Δ* cells, however, the staining was more confined to small vacuoles and in those cells with large vacuoles, the signal on the vacuolar membrane was only weak or even absent and staining was restricted to prevacuolar endosomes, especially in the *pho80Δ* strain (Fig 4A). This again suggests that the Pho85-Pho80 CDK-cyclin pair is required for an optimal fusion of endosomes with the vacuole.

Next, we evaluated the phosphorylation of two additional TORC1 clients to compare this with the phosphorylation of Sch9. The first client is Atg13, a regulatory subunit of the Atg1 complex involved in macroautophagy that was previously found to be phosphorylated at Ser^554^ by TORC1 localized on signaling endosomes [61,62]. We genomically expressed Atg13 as C-terminal triple HA-tagged fusion and quantified the phosphorylation-dependent band shift when using the anti-HA antibody. This demonstrated a consistent and significantly reduced phosphorylation of Atg13-HA_3_ in the *pho85Δ* and *pho80Δ* strains as compared to the WT strain (Fig 4B). Thus, similar as for Sch9, the Pho85-Pho80 CDK-cyclin pair has a direct or indirect effect on Atg13 phosphorylation, which may not be surprising because Pho85-Pho80 was shown to contribute to the complex regulation of autophagy when cells suffer nutrient starvation [44,63]. Of note, Atg13 was shown to recruit the phosphatidylinositol 3-kinase complex subunit Atg14 to the pre-autophagosomal structure in a phospho-dependent manner [64], and thereby Atg13 likely sets the conditions for the recruitment of Fab1 as well.

The second additional TORC1 client tested is Lst4, which in complex with Lst7 functions as GAP for the Rag family GTPase Gtr2 of the EGO complex [65]. At the vacuole, Lst4 ensures a rapid amino acid-dependent activation of TORC1, but once activated, TORC1 in turn phosphorylates Lst4 at several residues thereby triggering displacement of Lst4 from the vacuole. This feedback cycle prevents hyperactivation of TORC1 and safeguards the dynamic adjustment of TORC1 activity in response to amino acid availability [66]. We genomically expressed Lst4 as C-terminal V5-tagged fusion in the WT, *pho85Δ*, and *pho80Δ* strains and monitored the TORC1-dependent phosphorylation of Ser^523^. However, neither immunodetection with the anti-phospho-Lst4^S523^ antibody, nor the band shift seen when using the anti-V5 antibody, pointed to a significant difference in Lst4 phosphorylation between the strains (Fig 4C). Thus, even the *pho85Δ* and *pho80Δ* strains maintain sufficient vacuolar TORC1 activity to provide homeostatic control of Lst4. As such, it is unlikely that the reduced phosphorylation of Sch9 seen in these two deletion strains would solely be the consequence of a hampered vacuolar TORC1 recruitment, which raises again the possibility that Sch9 could be a specific substrate of the Pho85-Pho80 CDK-cyclin pair.

**Fig 5:**
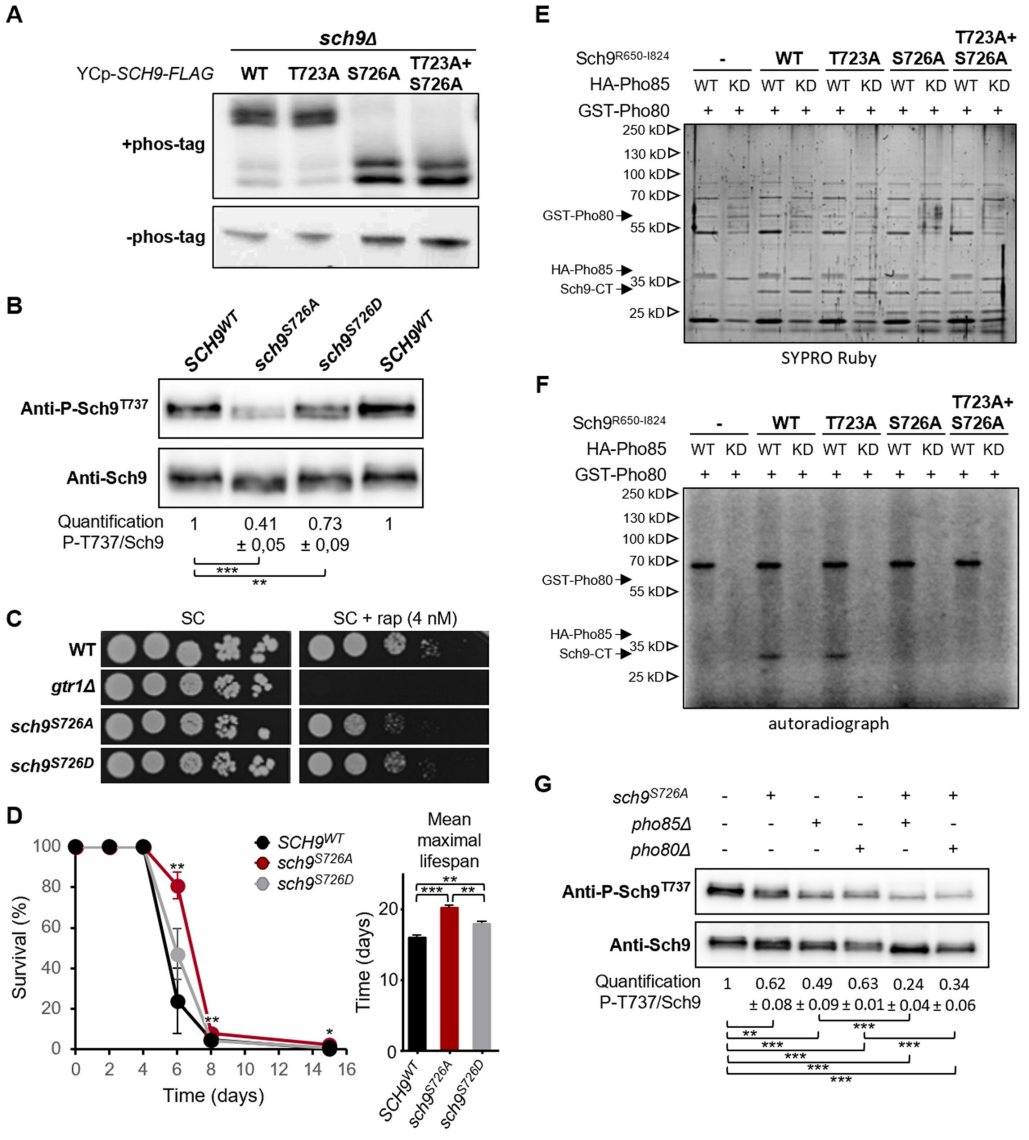
Pho85-Pho80-mediated phosphorylation of Ser^726^ primes Sch9 for its subsequent activation by TORC1. (A) Phos-tag immunoblot analysis of protein extracts obtained from exponentially growing *sch9Δ* cells transformed with a centromere plasmid driving the expression of C-terminally FLAG-tagged Sch9^WT^, Sch9^T723A^, Sch9^S726A^, or Sch9^T723A/S726A^ as indicated. Total protein extracts were resolved on phos-tag gels and were subsequently analyzed via immunoblotting with anti-FLAG antibodies. (B) Immunoblot analysis to assess the Sch9-Thr^737^ phosphorylation levels in protein extracts obtained from exponentially growing BY4741 cells expressing endogenous Sch9^WT^ or the mutant versions Sch9^S726A^ or Sch9^S726D^. The Thr^737^ phosphorylation levels were quantified based on the ratio of the signals obtained with the anti-P-Sch9^T737^ and anti-Sch9 antibodies and normalized to the ratio obtained for Sch9^WT^. (C) Rapamycin sensitivity analysis of BY4741 cells expressing endogenous Sch9^WT^ or the mutant versions Sch9^S726A^ or Sch9^S726D^ as assessed by growth on complete synthetic medium without or with 5 nM rapamycin. (D) Chronological lifespan assay showing the survival of BY4741 cells expressing endogenous Sch9^WT^ or the mutant versions Sch9^S726A^ or Sch9^S726D^ when starved for phosphate for several days as indicated. The bar diagram depicts the mean maximal lifespan. (E, F) Pho85-Pho80 phosphorylates Ser^726^ in the Sch9 C-terminus (CT). Purified recombinant Sch9^R650-I824^-TAP fragments corresponding to Sch9^WT^, Sch9^T723A^, Sch9^S726A^, or Sch9^T723A/S726A^ were subjected to *in vitro* phosphorylation by the Pho85-Pho80 CDK-cyclin pair purified from yeast. The assay was performed using wild-type Pho85 (WT) or the kinase dead Pho85^E53A^ mutant (KD), which was included as control. Representative SYPRO Ruby staining (E) and autoradiography (^32^P) blots (F) are shown. (G) Western blot analysis to assess the Sch9 Thr^737^ phosphorylation levels in protein extracts obtained from exponentially growing WT, *pho85Δ*, or *pho80Δ* cells expressing endogenous Sch9^WT^ or the mutant version Sch9^S726A^. The Thr^737^ phosphorylation levels were quantified based on the ratio of the signals obtained with the anti-P-Sch9^T737^ and anti-Sch9 antibodies and normalized to the ratio obtained for Sch9^WT^ in the WT strain. A two-tailed student’s T test was used to calculate significances (*, P < 0.1; **, P < 0.01; ***, P < 0.001).

Like the mammalian CDK counterparts, Pho85 is a proline-directed Ser/Thr protein kinase [35,67]. The previously reported TORC1 phospho-epitope mapping of Sch9 identified two Ser/Thr-Pro sites, *i*.*e*. Thr^723^ and Ser^726^ upstream of the hydrophobic motif (HM; amino acids 733–738) and, interestingly, this study suggested that the phosphorylation of Ser^726^ primed the kinase for Thr^723^ phosphorylation [1]. To confirm this and to address whether such a priming role would extend to other Sch9 phosphosites as well, we again turned to Phos-tag mobility shift assays, this time using Flag-tagged Sch9 constructs in which Thr^723^ and Ser^726^ were replaced by Ala, either separately or in combination. As shown in Fig 5A, only when Ser^726^ was replaced by Ala, the corresponding Sch9 mutants fail to become fully phosphorylated by TORC1, yielding a migration profile comparable to that seen for Sch9^WT^ and Sch9^5A^ in the *pho85Δ* strain (Fig 1D). We also tested other Sch9 phosphomutants, but only the Sch9^S726A^ was compromised in priming for subsequent TORC1-mediated phosphorylation (S4A Fig). To confirm this priming effect, we independently created genomic Sch9-Ser^726^-to-Ala and phosphomimetic Sch9-Ser^726^-to-Asp mutations and then tested the expressed proteins (*i*.*e*. Sch9^S726A^ and Sch9^S726D^) for their phosphorylation of Thr^737^ *in vivo*. Consistent with a priming effect, Thr^737^ phosphorylation was strongly reduced on Sch9^S726A^ when compared to the respective phosphorylation on Sch9^WT^ (Fig 5B). Notably, Thr^737^ was also slightly reduced on Sch9^S726D^, which indicates that the phosphomimetic Ser^726^-to-Asp mutation does not completely reproduce the changes seen by protein phosphorylation. We corroborated these data with growth assays where Sch9^S726A^-expressing cells were clearly rapamycin-sensitive, while Sch9^S726D^-expressing cells, although exhibiting a higher sensitivity to rapamycin than WT cells, were still coping better with rapamycin than Sch9^S726A^-expressing cells (Fig 5C). A similar picture was seen when we determined longevity, which is inversely correlated with Sch9 activity [68]. Accordingly, under phosphate starvation conditions, cells expressing the phosphomutant Sch9^S726A^ displayed a longer lifespan than those expressing Sch9^WT^, with cells expressing Sch9^S726D^ exhibiting a lifespan that was between the ones of Sch9^WT^ and Sch9^S726A^ expressing cells (Fig 5D). No significant difference in lifespan was seen between these cells when starved for nitrogen or carbon (S4D Fig), which was to be expected since both conditions abrogate the TORC1-dependent phosphorylation of Sch9 [69].

To test whether the Pho85-Pho80 CDK-cyclin pair directly phosphorylates Sch9, we next performed an *in vitro* protein kinase assay. To this end, HA-Pho85, the kinase-inactive HA-Pho85^E53A^, and GST-Pho80 were purified from yeast lysates and peptides covering the C-termini of Sch9^WT^, Sch9^T723A^, Sch9^S726A^, or Sch9^T723A,S726A^ were used as substrates. As shown, phosphorylation was obtained with the peptides corresponding to Sch9^WT^ and Sch9^T723A^, but not with those corresponding to Sch9^S726A^ or Sch9^T723A,S726A^, thus confirming that Ser^726^ is indeed the predominant epitope phosphorylated by Pho85-Pho80 (Figs 5E, 5F and S4B). When combined, our data thus corroborate that phosphorylation of Sch9 on Ser^726^ by Pho85-Pho80 primes Sch9 for its phosphorylation by TORC1.

As shown above, both Tor1 and Sch9 are still present at membranes of emerging small vacuoles in the *pho85Δ* and *pho80Δ* strains, but this localization is hampered as vacuoles become larger, especially in the *pho80Δ* strain. In consequence, these small vacuoles must be the primary site where TORC1-mediated Sch9 activation occurs in both deletion strains. Since vacuolar size is inversely correlated to Fab1 activity [59], our data also infer a low PI[3,5]P2 content in the membranes of the enlarged vacuoles, which is consistent with our observation that the Pho85-Pho80 CDK-cyclin pair plays a role to properly shift Fab1 from signaling endosomes to vacuoles. To get an estimate on the relative contributions to Sch9-Thr^737^ phosphorylation through both the Fab1-mediated TORC1 control and the Pho85-Pho80-mediated Sch9-Ser^726^ priming phosphorylation, we first sought to rule out that the phosphorylation of Sch9 at Ser^726^ itself affects the vacuolar recruitment of Sch9. In a previous report it was already shown that the GFP-Sch9^5A^ and GFP-Sch9^2D3E^ mutants, which both include the Ser^726^mutation, normally localize to the vacuolar membranes when expressed in WT cells [1]. To elaborate on this, we examined the intracellular localization of GFP-Sch9^S726A^ and GFP-Sch9^S726D^ and found, as expected, both fusion proteins to normally localize at the vacuole as well (S4C Fig). Next, we studied whether the reduced phosphorylation level of Thr^737^ on the priming site mutant Sch9^726A^ would be further reduced by loss of Pho85 or Pho80. This was indeed the case as the partially compromised Sch9-Thr^737^ phosphorylation levels in *pho85Δ* and *pho80Δ* cells were further reduced by roughly 50% when combined with the Sch9^S726A^ mutation (Fig 5G). These data combined not only indicate that priming of Sch9 at Ser^726^ and proper regulation of Fab1 are almost equally important for optimal activation of Sch9 by TORC1, but also that Ser^726^ can be targeted by other kinases as well. The latter fits well with another recent report in which Sch9-Ser^726^ has been suggested to be phosphorylated by the CDK9 homologue Bur1 [70].

### Pho85-Pcl6 and Pho85-Pcl7 differentially impact on Sch9 phosphorylation

The observation that episomal expression of Sch9^2D3E^ partially alleviated the rapamycin sensitivity of the *pho80Δ* strain, but not that of the *pho85Δ* strain, suggested that additional cyclins are involved in mediating a normal activation of Sch9 by TORC1. The best-placed candidates to make such a contribution would be Pcl6 and Pcl7, because loss of both proteins together led to a severe synthetic growth defect when combined with loss of Sch9 (Figs 1A and S1). It is well established that both Pho85-Pcl6 and Pho85-Pcl7 contribute to the regulation of the type1 protein phosphatase Glc7 via control of its regulatory subunit Glc8, but, while Pho85-Pcl7 is the best performing kinase *in vitro*, Pho85-Pcl6 is the main Glc8 kinase *in vivo* [71]. We examined the possibility that Glc7 may dephosphorylate Sch9 by monitoring Sch9-Thr^737^ phosphorylation in the *pcl6Δ*, *pcl7Δ*, and *pcl6Δ pcl7Δ* strains during exponential growth. We noted that the Sch9 phosphorylation level was not affected in the *pcl6Δ* strain but reduced in the *pcl7Δ* strain as compared to the WT strain (S5A Fig). In addition, we also examined strains lacking Glc8, or other non-essential Glc7-interacting proteins, using the approach previously described that identified Glc7-Shp1 as a protein phosphatase for Rps6 [72]. Although we observed some variation among the strains, none of them maintained significant Sch9 phosphorylation levels after rapamycin treatment (S5B Fig), suggesting that the Glc7 phosphatase does not play a major role in controlling the Sch9 phosphorylation status under the conditions tested.

**Fig 6:**
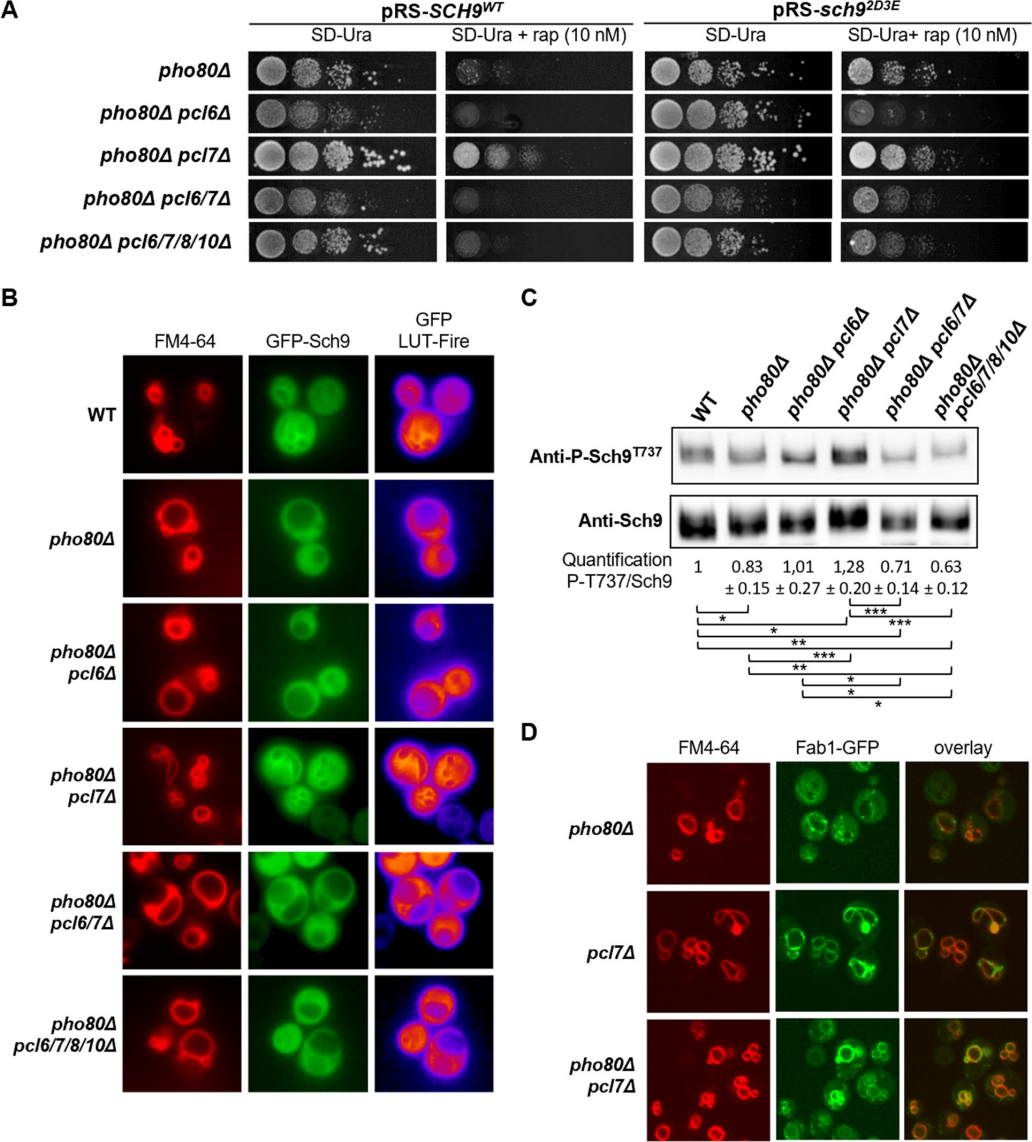
The Pho85-cyclins Pcl6 and Pcl7 contribute to the regulation Fab1 and the vacuolar recruitment of Sch9. (A) Rapamycin sensitivity analysis of the *pho80Δ*, *pho80Δ pcl6Δ*, *pho80Δ pcl7Δ*, *pho80Δ pcl6Δ pcl7Δ*, and *pho80Δ pcl6Δ pcl7Δ pcl8Δ pcl10Δ* strains expressing either Sch9^WT^ or Sch9^2D3E^ from a centromere plasmid. The strains were spotted on selective synthetic medium without and with 10 nM rapamycin. (B) Microscopic analysis of the strains mentioned in (A) but expressing GFP-Sch9^WT^. The strains were grown to mid-log phase on selective synthetic medium. The lipophilic dye FM4-64 was used to visualize the vacuolar membrane. (C) Immunoblot analysis of protein extracts from the strains mentioned in (A) and exponentially grown on complete synthetic medium to assess changes in Sch9 phosphorylation. The Sch9-Thr^737^-phoshorylation levels were quantified based on based on the ratio of the anti-P-Sch9^T737^ and anti-Sch9 signals, and normalized to the ratio obtained for the WT cells. A two-tailed student’s T test was used to calculate significances (*, P < 0.1; **, P < 0.01; ***, P < 0.001). (D) Microscopic analysis of Fab1-GFP localization in the *pho80Δ*, *pcl7Δ*, and *pho80Δ pcl7Δ* strains. The strains were grown to mid-log phase on selective synthetic medium. The lipophilic dye FM4-64 was used to visualize the vacuolar membrane.

This led us to use another strategy and to combine the *PHO80* deletion with combinations of the *PCL6* and *PCL7* deletions. We also created the quintuple *pho80Δ pcl6Δ pcl7Δ pcl8Δ pcl10Δ* deletion mutant that lacks all Pho80-like cyclin subfamily members to serve as an additional control. The strains were again tested for their rapamycin sensitivity when expressing Sch9^WT^ or Sch9^2D3E^ from centromere plasmids and this revealed an intricate interplay of the cyclins. Indeed, when compared to the *pho80Δ* strain, the additional deletion of *PCL6* prevented Sch9^2D3E^ from rescuing the rapamycin-induced growth defect, while the deletion of *PCL7* improved growth both of the Sch9^WT^ and Sch9^2D3E^ transformants and under conditions with or without rapamycin addition. However, it sufficed to introduce the *PCL6* deletion in the *pho80Δ pcl7Δ* strain to abrogate its improved growth (Fig 6A). The quintuple control strain behaved like the *pho80Δ pcl6Δ* and the *pho80Δ pcl6Δ pcl7Δ* strains, confirming that Pcl8 and Pcl10 did not contribute to the observed phenotype. Consistently, a similar phenomenon was seen when monitoring the vacuolar size and vacuolar membrane recruitment of GFP-tagged Sch9^WT^. In contrast to the loss of Pcl6, the loss of Pcl7 prevented the formation of enlarged vacuoles with reduced Sch9 decoration that typifies the *pho80Δ* strain, but again this phenotype was reverted when the *pho80Δ* strain lacked both Pcl6 and Pcl7 (Figs 6B and S5C). Furthermore, we observed a significantly increased Sch9-Thr^737^ phosphorylation in the *pho80Δ pcl7Δ* strain as compared to the single *pho80Δ* strain and the WT strain. However, in the *pho80Δ pcl6Δ pcl7Δ* and the control strain the degree of Sch9-Thr^737^ phosphorylation was again markedly reduced (Fig 6C). This suggests that Pho85-Pcl6 and Pho85-Pcl7 may oppositely impact on the activity of the Fab1 complex, either on Fab1 itself or on one of its regulatory subunits, *i*.*e*. Fig4, Vac14, Vac7, or Atg18 [20].

Since both vacuolar size and vacuolar recruitment of Sch9 are linked to PI[3,5]P_2_ production, and given that vacuolar recruitment is a prerequisite for the phosphorylation of Sch9 by TORC1, we wondered whether Fab1 would localize again at the vacuolar membrane in the *pho80Δ pcl7Δ* strain. To this end, we transformed the *pcl7Δ* and *pho80Δ pcl7Δ* strains with the aforementioned centromere *FAB1-GFP* or *fab1*^*VLA*^*-GFP* plasmids and compared the expression levels and localization of both fusion proteins with that seen in the *pho80Δ* strain. The additional deletion of *PCL7* in the *pho80Δ* cells partially restored the expression levels of both fusions (S5D Fig). Furthermore, Fab1-GFP nicely stained the vacuolar membranes in both the *pcl7Δ* strain and the *pho80Δ pcl7Δ* strain, indicative that the additional deletion of *PCL7* in the *pho80Δ* strain also restored the shift of Fab1 from endosomes to the vacuolar membrane (Fig 6D). The Fab1^VLA^-GFP expression, however, came along with tiny vesicle-like vacuoles and, similar as seen for all other strains tested (Fig 2E), Fab1^VLA^-GFP still localized in perivacuolar foci in the *pcl7Δ* and *pho80Δ pcl7Δ* cells, which we believe to correspond to endosomes (S5E Fig). As such, these data indicate that even in the *pho80Δ pcl7Δ* strain the hyperactive Fab1 mutant inherently fails to properly translocate from endosomes to vacuoles and to feed vacuolar membrane biogenesis.

Of note, the phenotypes observed for the *pho80Δ pcl7Δ* strain most closely resembled those described above for the *pho81Δ* mutant (Figs 1B, 1E, 2D and 2E)). This is not surprising given that Pcl7 and Pho80 are the only known members of the Pho80-like cyclin family that physically interact with Pho81 [25,28,29,73]. Furthermore, similarly as for the *pho81Δ* strain (Fig 1A), tetrad analysis confirmed that also in case of the *pho80Δ pcl7Δ* strain the presence of Sch9 is essential to maintain growth (S1 Fig).

### Identification of Pho4 as effector for Pho85 and TORC1-Sch9 crosstalk

**Fig 7:**
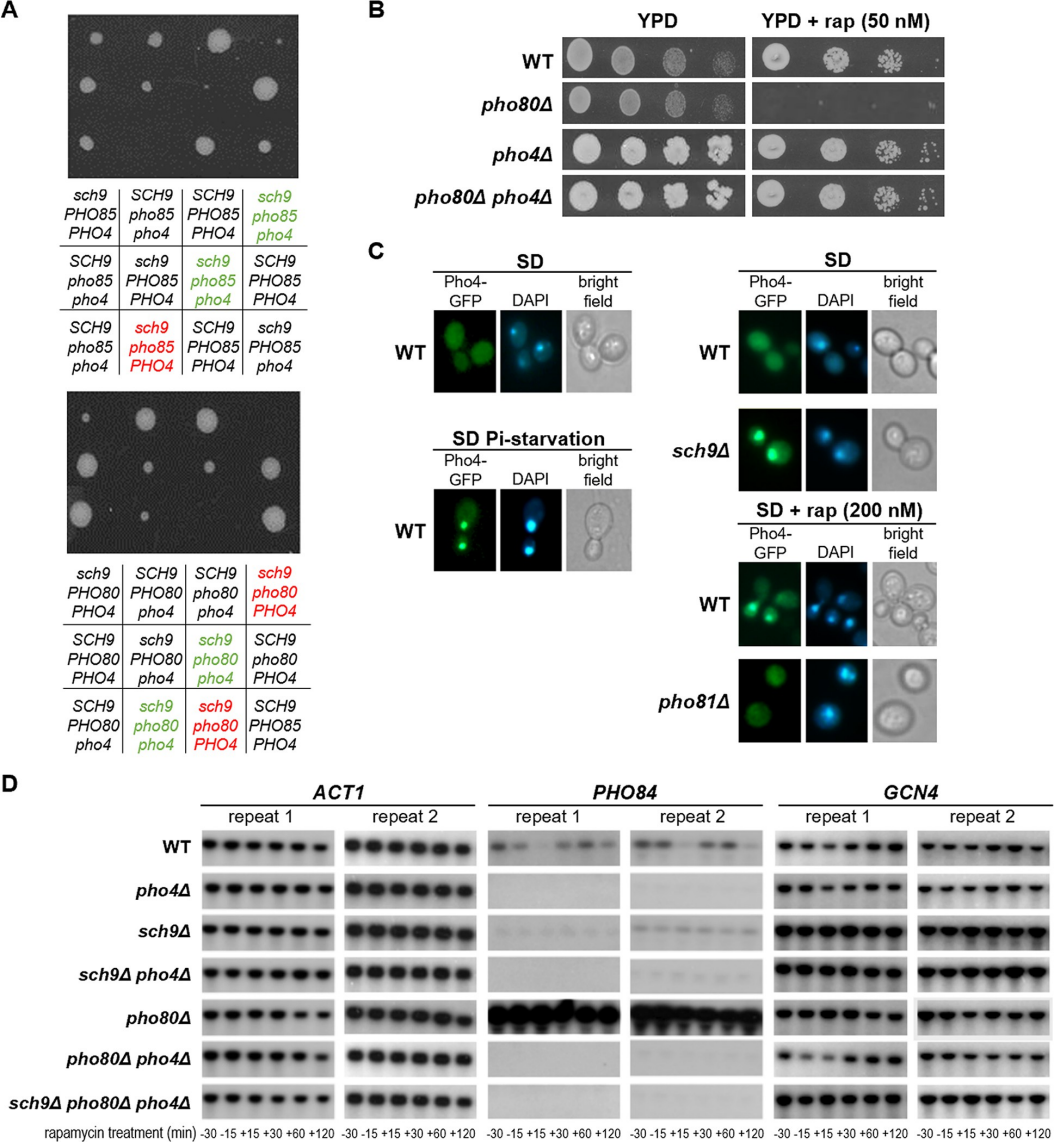
The transcription factor Pho4 is a mutual target Pho85 and Sch9. (A) Tetrad analysis demonstrated that the additional deletion of *PHO4* rescues the synthetic lethality of the *pho85Δ sch9Δ* and *pho80Δ sch9Δ* strains (see genotypes indicated in green and red, respectively). (B) Rapamycin sensitivity analysis of the WT, *pho80Δ*, *pho4Δ*, and *pho80Δ pho4Δ* strains spotted on YPD plates without and with 50 nM rapamycin (rap). (C) Microscopic analysis of Pho4-GFP localization in WT, *sch9Δ*, and *pho81Δ* cells. Cells were grown to mid-log phase in selective synthetic medium. As indicated, part of the WT and *pho81Δ* cultures were then either washed and transferred to phosphate starvation medium or subjected to 200 nM rapamycin treatment for 2 hours. The cells were stained with DAPI to visualize the nucleus. (D) Northern blot analysis to monitor the expression of *PHO84*, *GCN4*, or the *ACT1* control in the WT, *pho4Δ*, *sch9Δ*, *sch9 pho4Δ*, *pho80Δ*, *pho80Δ pho4Δ*, and *sch9Δ pho80Δ pho4Δ* strains before or after treatment with 200 nM rapamycin for the time indicated.

To further clarify the mechanisms by which dysfunction of Pho85 signaling is leading to a synthetic lethality in the *sch9Δ* background, we next tested the contribution of three well-known downstream targets of the Pho85-Pho80 CDK-cyclin pair, *i*.*e*. the protein kinase Rim15 and the transcription factors Crz1 and Pho4 [31,34,43,67,74]. To this end, we crossed the *RIM15*, *CRZ1*, or *PHO4* deletion into the *pho85Δ* and *pho80Δ* strains and then mated these with the *sch9Δ* strain. Tetrad analysis showed that the deletion of *PHO4*, but not the deletion of *RIM15 or CRZ1*, allowed outgrowth of the triple deletion spores, but, while the loss of Pho4 alleviated the synthetic lethality of the combined *SCH9* and *PHO80* deletion, the germinated *sch9Δ pho85Δ pho4Δ* spores were still severely sick as the strains grew very poorly and lost viability after storage (Figs 7A and S6A, S6B). Interestingly, loss of Pho4 also rescued the rapamycin-induced growth inhibition of the *pho80Δ* mutant (Fig 7B). These data suggest that inappropriate Pho4-mediated transcription could be the cause for the observed synthetic lethality when signaling through Pho85-Pho80 and the TORC1-Sch9 axis is deregulated.

Pho4 controls the transcription of genes in response to phosphate starvation but in phosphate-rich medium it is phosphorylated by Pho85-Pho80 and excluded from the nucleus [67,75] (Fig 7C). We analyzed whether TORC1 and Sch9 would affect the intracellular localization of Pho4 by expression of a C-terminally tagged Pho4-GFP version. As shown, the Pho4-GFP fusion protein localized in the cytoplasm in exponentially growing WT cells, but it translocated into the nucleus when the WT cells were subjected to phosphate starvation. Interestingly, Pho4-GFP also localized in the nucleus when WT cells were treated with rapamycin as well as in exponentially growing *sch9Δ* cells, which suggests that the TORC1-Sch9 axis controls the nuclear import of the transcription factor. Yet, Pho4-GFP remained cytoplasmic in rapamycin-treated *pho81Δ* cells, indicative that the rapamycin treatment did not overrule the regulation of Pho4 by Pho85-Pho80 (Fig 7C). In addition, we performed Northern blot analysis to monitor the expression of *PHO84*, encoding the high-affinity phosphate transporter. As expected, the transcription of *PHO84* was clearly upregulated in the *pho80Δ* strain in a Pho4-dependent manner but despite the nuclear translocation of Pho4 upon rapamycin treatment, or the deletion of *SCH9*, no induction of *PHO84* was observed under these conditions (Fig 7D). On the contrary, the addition of rapamycin triggered *PHO84* repression with a transient recovery up to 60 min, while the deletion of *SCH9* resulted in a constitutive low basal expression of *PHO84*. Similar results were obtained when we used RT-PCR to monitor the expression of *PHO5*, another Pho4-dependent gene encoding an acid phosphatase (S6C Fig). Thus, even though Pho4 resides in the nucleus in rapamycin-treated WT and *sch9Δ* cells, neither *PHO84* nor *PHO5* were induced. Whether this is due to a deregulation of Pho4 or one of the auxiliary transcription factors required for expression of the PHO regulon [76], remains to be clarified. Another process that should be considered is chromatin remodeling as this is known to be controlled by the TORC1-Sch9 axis via Ino80 and required for opening the chromatin at the promotors of several metabolic genes, including *PHO5* [77,78].

Since Pho4 has been shown to fine-tune the timely transcription of post-diauxic genes that are also responsive to amino acid starvation [79], we additionally monitored the expression of a known Sch9 target, *i*.*e*. the transcription activator of the general amino acid control pathway encoded by *GCN4* [5]. We have chosen *GCN4* because it controls a major number of amino acid biosynthesis and nitrogen responsive genes [80] and because the stability of this transcription factor under amino acid starvation conditions is stringently controlled Pho85 signaling [29], thus providing an interesting point of crosstalk with TORC1-Sch9 signaling. Consistent with our previously published data [5], the *sch9Δ* strain already displayed an enhanced expression of *GCN4* during exponential growth and this level was maintained during the rapamycin treatment (Fig 7D). Enhanced *GCN4* expression was also observed in the *pho80Δ* strain, which was expected given that Pho85-Pho80 phosphorylation primes Sch9 for full TORC1-mediated activation. However, while the derepression in the *sch9Δ* mutant appeared to be Pho4 independent, that of the *pho80Δ* mutant was clearly mediated by Pho4 as the *GCN4* expression profile in the *pho80Δ pho4Δ* strain was more comparable to that of the WT strain. Yet, the additional deletion of *SCH9* in the *pho80Δ pho4Δ* strain rendered *GCN4* derepression again Pho4-independent (Fig 7D). These data indicate that the loss of Sch9 overrules the Pho4 requirement thereby defining Sch9 as direct regulator of *GCN4* expression and suggesting that the role of Pho4 is restricted to fine-tuning via the PHO pathway. As such, it would be interesting to analyze whether this Pho4-dependent fine-tuning of *GCN4* is mediated by the enhanced expression of Pho84 and signaling via Pho81.

## Discussion

In this paper, we aimed to understand how two distinct kinases, Pho85 and TORC1, control nutrient signaling [11,43]. That both loss of Pho85 or its inhibitor Pho81 result in a synthetic growth defect when combined with Sch9 deletions not only indicates a close link to phosphate sensing, but it shows that growth is depending on a tight balance between Pho85 and TORC1-Sch9 signaling. We found this relation between Pho85 and TORC1-Sch9 in supporting growth to be multifaceted, involving Pho80 and the partially redundant cyclins Pcl6 and Pcl7.

### Events occurring at the vacuole and signaling endosomes

**Fig 8:**
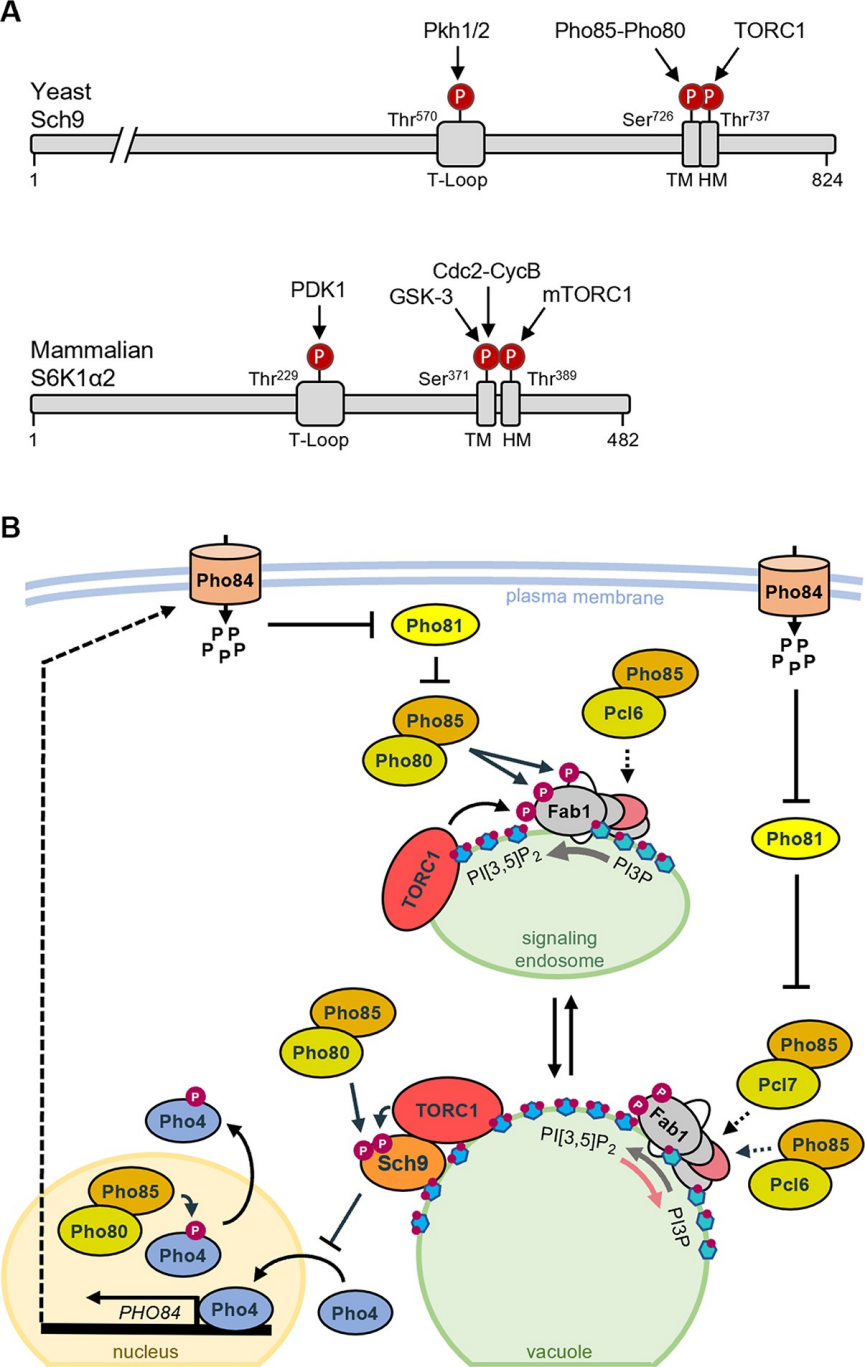
Crosstalk between the PHO pathway and TORC1-Sch9 signaling and conservation of the turn motif priming principle. (A) Schematic representation of yeast Sch9 and mammalian S6K1 to highlight the evolutionary conservation of the TM priming principle. Indicated are the phosphorylation sites within the T-loop, which is targeted by Pkh1/2 in Sch9 and PDK1 in S6K1, and within the turn motif (TM) that is targeted by Pho85-Pho80 in Sch9 and by GSK3 and Cdc2-CycB in S6K1 as well as the phosphorylation site in the C-terminal hydrophobic motif that is targeted by TORC1 in Sch9 and mTORC1 in S6K1. (B) Shown is a hypothetical model based on previously reported data and observations made in our current study to depict the possible different connections for the interplay between the PHO pathway and the TORC1-Sch9 axis. At the endosome, TORC1 and Pho85-Pho80 phosphorylate and stimulate the Fab1 lipid kinase subunit to convert PI3P into PI[3,5]P_2_ (grey arrow) [22,50]. Pho85-Pho80 also phosphorylates Vac7 (white subunit) [50], thereby probably enhancing endosomal fusion. Pho85-Pcl6 could act on the Vac14-Fig4 subcomplex to induce the protein phosphatase activity of Fig4 (pink subunit) required to relieve an inhibitory autophosphorylation of the Fab1 subunit [87]. At the vacuole, Sch9 is recruited by binding PI[3,5]P_2_ where it is phosphorylated by Pho85-Pho80, which primes Sch9 for its subsequent phosphorylation and activation by TORC1. In the Fab1 complex, Pho85-Pcl7 may act on the Vac14-Fig4 subcomplex to control the lipid phosphatase activity of Fig4, which allows to recycle PI3P from PI[3,5]P_2_ (pink arrow) [57,58] Active Sch9 prevents nuclear entry of the Pho4 transcription factor, while phosphorylation of Pho4 by Pho85-Pho80 triggers its export from the nucleus [34,67], thereby preventing transcription of *PHO84*, which encodes a high-affinity phosphate transporter [88]. Phosphate uptake by Pho84 inhibits Pho81, which in turn acts as inhibitor for Pho85-Pho80 and Pho85-Pcl7 [28,73].

It is well known that TORC1 phosphorylates and activates Sch9 at the vacuolar membrane [1,22,52]. We now provide evidence that Pho85-Pho80 directly phosphorylates Sch9 at Ser^726^ and that this primes Sch9 for further activation by TORC1. The priming effect of phospho-Ser^726^ has previously been noticed for the nearby epitope Thr^723^ when studying TORC1-dependent Sch9 phosphorylation [1], but now we extend these data by showing that Ser^726^ is targeted by Pho85-Pho80 and that its priming effect also applies to other TORC1-dependent epitopes, such as Thr^737^. Intriguingly, Ser^726^ is located immediately adjacent to the C-terminal hydrophobic motif (HM; that contains Thr^737^), and a similar priming site for subsequent mTORC1-mediated HM phosphorylation has been described for mammalian S6K1 (Fig 8A). In this case, the S6K1-Ser^371^, like the Sch9-Ser^726^, is also followed by a proline and phosphorylated by proline-directed kinases including Cdc2-cyclin B and GSK-3 [81–84]. Notably, the Ser^371^-Pro-^372^ motif in S6K1 has been coined turn motif (TM), which occurs in some AGC kinases where it stabilizes the kinase and/or promotes the phosphorylation state within the HM motif when it is phosphorylated at the Ser position [85,86]. Based on the functional analogy and structurally similar positioning of S6K1-Ser^371^ and Sch9-Ser^726^ just upstream of the HM, we therefore infer that the Ser^726^-Pro^727^ motif in Sch9 corresponds to the TM and that TM priming sites represent an evolutionary conserved principle that allows S6K1 and Sch9 to integrate additional signals (Fig 8A).

The fact that Pho85-Pho80 phosphorylate Ser^726^ implies that the full activation of Sch9 requires phosphate uptake and phosphate sensing via the CDK-inhibitor Pho81, which is consistent with the observed increased Sch9 phosphorylation in the *pho81Δ* strain. It is also consistent with the observation that, particularly under phosphate starvation conditions, cells expressing the phosphomutant Sch9^S726A^ display a longer lifespan as compared to cells expressing the phosphomimetic variant Sch9^S726D^ or the Sch9^WT^ allele. We were not the first to notice a connection to phosphate signaling. A recent study demonstrated a link between phosphate acquisition via the high-affinity transporter Pho84 and TORC1 activity as assayed by Sch9 phosphorylation [89]. The authors proposed the Gtr1 Rag-GTPase of the EGO complex as the main phosphate signal receiver upstream of TORC1, thus acting in parallel to Pho85-Pho80. Given that Sch9 is an orthologue of PKB/Akt1, it is interesting to note that a similar role of phosphate has been noticed in mice where a high phosphate diet activates the Akt-mTORC1-S6K pathway thereby accelerating aging [90].

Pho85 signaling also elicits a second important effect that equally contributes to the regulation of the TORC1-Sch9 signaling axis. Just like TORC1 [22], Pho85 signaling controls the distribution of Fab1 between endosomal and vacuolar membranes as well. As such, Pho85 and its cyclins impact on the PI[3,5]P_2_ content in the vacuolar membrane, which in turn is required for the recruitment of Sch9 and essential for its TORC1-dependent phosphorylation [21]. We show that mainly the Pho80 and Pcl7 cyclins are at play here. Albeit control of PI[3,5]P_2_ synthesis by Pho85 signaling has been described as a stress response [50], it is clear that it is also required to maintain an optimal PI[3,5]P_2_ synthesis under non-stressed conditions as evidenced by the observation that a significant fraction of *pho85Δ* and *pho80Δ* cells display enlarged vacuoles while *pho81Δ* and *pcl7Δ* cells appear to have more fragmented small vacuoles during exponential growth. The question is thus how the Pho85 signaling interferes with the endosomal and vacuolar distribution of Fab1. Our data on Fab1-GFP and Fab1^VLA^-GFP suggest that besides controlling the activity of Fab1 itself [50], Pho85-Pho80 also ensures an optimal fusion of signaling endosomes and an optimal recycling of Fab1 from the vacuolar membrane. To understand these actions, one must look at the Fab1 complex. Apart from its N-terminal FYVE domain and the C-terminal lipid kinase domain, the central region of the Fab1 subunit harbors two additional conserved domains, *i*.*e*. the CCT-like domain that shares homology with ‘chaperonin-containing TCP-1’ chaperonins and the cysteine-rich domain. These domains allow binding of Fab1 to the Vac14 and Fig4 subunits. Vac14 is a scaffold that coordinates the activities of Fab1 and Fig4, the latter being a PI[3,5]P_2_-5-phosphatase, which in addition has protein phosphatase activity that counteracts a repressive autophosphorylation in the kinase activation loop of Fab1 [20,87]. The scaffold Vac14 also interacts with the regulatory subunits Atg18 and Vac7. Both these subunits are particularly important. Atg18, also known as Svp1, binds PI3P at the pre-autophagosomal structure and endosomes but PI[3,5]P_2_ at the vacuole where it fulfils an important role in membrane recycling from the vacuole to late endosomes [20,56,91,92]. Consistently, we have previously shown by using a fluorescent PI[3,5]P_2_-reporter that deletion of *ATG18* shifts this reporter from the signaling endosome to the vacuole, which indeed suggests that Atg18 is involved in the recycling of Fab1 complex from the vacuole to the signaling endosome [22]. Atg18 requires Vac7 for its recruitment at the vacuolar membrane [20,56]. Vac7 is a transmembrane protein and positive regulator of the Fab1 complex that is phosphorylated by Pho85-Pho80 [20,50]. Recently, it was shown that Vac7 shares a late embryogenesis abundant-2 (LEA) domain with Tag1, a protein named after its role to terminate autophagy, which predicts that both Vac7 and Tag1 are important for lipid transfer [93]. This raises the possibility that phosphorylation of Vac7 by Pho85-Pho80 facilitates endosomal fusion to deliver PI[3,5]P2 and the Fab1 complex to the vacuole as depicted in Fig 8B. Concerning the role of Pho85-Pcl7, our data suggest that it mainly impacts on the vacuolar PI[3,5]P_2_ content and vacuolar fission. Hence, a possible scenario would be that Pho85-Pcl7 reduces the PI[3,5]P_2_ levels by placing the Fab1 complex in a configuration that favors the Fig4 lipid phosphatase activity to convert PI[3,5]P_2_ back to PI3P at the vacuole [57,58]. As such, Pho85-Pcl7 would either target Fig4 or Vac14, because Fig4 not only needs to be recruited by Vac14 to the Fab1 complex, but it also must interact with the Vac14 scaffold to be active [57,87]. Finally, our data also strongly suggest that the Pho85-Pcl6 CDK-cyclin pair opposes the role of Pho85-Pcl7 in controlling the Fab1 complex. This became most obvious by the observation that the loss of Pcl7 no longer prevented the formation of enlarged vacuoles in the *pho80Δ pcl6Δ* mutant, while this was readily the case in the *pho80Δ* strain. Furthermore, the additional deletion of *PCL6* also hampered Sch9^2D3E^ to rescue the growth of the *pho80Δ* strain on rapamycin-containing medium, which is probably due to a further reduction of the PI[3,5]P_2_ levels and the vacuolar recruitment of Sch9^2D3E^ in the *pho80Δ pcl6Δ* strain. Thus, it may well be that Pho85-Pcl6 also impacts on the Vac14-Fig4 subcomplex, for instance, to enhance the protein phosphatase activity of Fig4 required to counteract the autophosphorylation of Fab1 at Ser^48^ and Ser^2053^, which repress the basal activity of the Fab1 subunit [87]. While at the moment this is only speculative, it is important to realize that the opposing roles of Pcl6 and Pcl7 were only observed in the *pho80Δ* background in which the phosphorylation of the Fab1 kinase and its regulator Vac7 are compromised [50]. Importantly, there are many other players involved in vacuolar fission/fusion, such as Env7 [94], the HOPS subunit Vps41 [95] or the I-BAR signature protein Ivy1 that seems to control the availability of PI3P for Fab1 at signaling endosomes [24,96]. Hence, further research is needed to fully understand the roles of Pho85 and the aforementioned cyclins in controlling the endosome-vacuole dynamics.

The fact that loss of Pcl7 counteracts the loss of Pho80 in controlling the vacuolar PI[3,5]P_2_ content and the recruitment of Sch9 is interesting since these are the two cyclins that interact with the CDK-inhibitor Pho81. It indicates that Pho81 fulfills a balancing role by adjusting the cellular PI[3,5]P_2_ content in function of phosphate availability. At least for Pho85-Pho80, the inhibitory action of Pho81 depends on myo-d-inositol heptakisphosphate or IP7 [25], underscoring the importance of inositol polyphosphate signaling to maintain the proper balance of PI[3,5]P_2_ levels and phosphate availability. Kcs1 is the main IP6 kinase in yeast and, not surprisingly, its deletion is synthetic lethal when combined with loss of Sch9 [11]. In line with this, we observed that a proper equilibration of the PI[3,5]P_2_ levels is not only essential for Pho85 signaling, but for TORC1 signaling as well, and that the activities of Fab1 and Sch9 must be aligned in order to support growth on rapamycin. This is consistent with the previously made observation that strains harboring a deletion of *FAB1* or the TORC1 phosphomimetic *fab1*^*6D*^ allele, both characterized by reduced Sch9 phosphorylation, are more rapamycin sensitive, this in contrast to a strain expressing the phosphomutant Fab1^6A^ in which the Sch9 phosphorylation level is similar as that in WT cells [22].

Another interesting aspect is that the expression of Pcl7 is cell cycle dependent and peaks in S-phase, while the expression of Pcl6 is constitutive [28]. This makes Pcl7 a prime candidate to control the PI[3,5]P_2_ levels during the course of the cell cycle. Indeed, the PI[3,5]P_2_-mediated process of vacuolar fission and fusion not only allows to adjust the vacuolar surface-to-volume ratio and the retrograde traffic from the vacuole to the Golgi upon environmental changes, but during the cell cycle vacuolar membrane fission is important for the transmission of the organelle to the growing daughter cell [97]. Obviously, also Pho85-Pho80 and TORC1 play their part in orchestrating the vacuolar fission/fusion equilibrium and cell cycle [22,31,50,51,97–99]. In connection to Sch9, it was shown that this kinase is only recruited to newly formed vacuoles at a late stage in their maturation process and that the TORC1-dependent phosphorylation of Sch9 then signals the vacuolar maturity to the cell cycle machinery, thereby dictating cell cycle progression [52]. Most recently, this link between vacuole maturation and cell cycle progression was further strengthened by showing that not only TORC1 is at play, but that in parallel Sch9 becomes phosphorylated by the CDK9 homologue Bur1. Based on mass spectrometry, the authors identified eleven Bur1-sensitive epitopes, including the Pkh1/2 phosphoepitope Thr^570^ as well as canonical CDK sites Thr^723^ and Ser^726^, the latter being the residues we associated with Pho85-Pho80 priming [70]. Bur1, also known as Sgv1, is an essential protein that was proposed to act in the same pathway as the G1 cyclin Cln3 [100]. It is mainly, but not exclusively, localized in the nucleus where it acts together with its cyclin Bur2 to modify histones, to control transcription, and to regulate telomere length [101]. The latter is of particular interest since telomere length is also known to be affected by the loss of Pho85-Pho80, Gtr1, or the catalytic (Vps34) and regulatory (Vps15) subunits of the PI3-kinase [102]. As such, it is tempting to speculate that especially telomere length may serve as additional checkpoint that is signaled to the cell cycle machinery by the phosphorylation of Sch9. An issue might be the nuclear localization of Bur1/Bur2. However, note that Pho80 can drag other proteins into the nucleus as shown for Pho81 and Fab1 [50,103]. Whether this is also the case for Sch9 is currently unknown as we did not observe a nuclear accumulation of GFP-Sch9 in our studies, but at least under hyperosmotic shock, which induces a temporary arrest of cell-cycle progression, Sch9 was reported to be nuclear and to act as chromatin-associated transcription activator of stress responsive genes [104,105].

To end, the role of Pho85 signaling in simultaneously controlling PI[3,5]P_2_ synthesis and priming Sch9 for activation by TORC1 may be conceptually conserved in higher eukaryotes. Accordingly, in neuronal cells, the Pho85-Pho80 orthologous CDK5-p35 complex directly phosphorylates S6K1 at Ser^411^ located within the autoinhibitory domain, thereby controlling dendritic spine morphogenesis, a process in which metabolic turnover and compartmentalization of phosphoinositides play an important role and where the CDK5-p39 complex controls the endosomal adaptor protein WD repeat and FYVE domain-containing 1 (WDFY1) [106–108]. Interestingly, here also Ser^411^ phosphorylation primes S6K1 for its subsequent rapamycin-sensitive phosphorylation of Thr^389^ and activation [109]. In insulin-stimulated adipocytes, Cdk5-dependent phosphorylation is not only observed for Ser^411^ but for Ser^424^ and Ser^429^ as well, and the latter appears to dictate altered S6K1 substrate specificity towards enzymes involved in lipid metabolism [110]. In addition, CDK5–p35 also phosphorylates mammalian Fab1/PIKfyve to positively regulate PI[3,5]P_2_ production [50] and in adipocytes enhanced PI[3,5]P_2_ levels are associated with the mTORC1-mediated stimulation of S6K1 [111]. Finally, CDK5 also shows significant crosstalk with the PI3K-Akt cascade in prostate cancer cell proliferation since CDK5 seems to physically interact with Akt to control Akt membrane sequestration and androgen receptor-mediated activation [112].

### Events happening in the nucleus

Apart from the processes occurring at the vacuole, we show that the synthetic lethality caused by an imbalanced Pho85 and TORC1 signaling is also associated with Pho4. This transcription factor is excluded from the nucleus due to its phosphorylation by Pho85-Pho80 when cells are growing in nutrient-rich medium with plentiful phosphate [34,113]. We now show that Pho4 is retained in the cytoplasm if Sch9 is active and that it translocates to the nucleus upon rapamycin treatment or loss of Sch9. This nucleocytoplasmic regulation is reminiscent to the control of Rim15, which is also excluded from the nucleus by phosphorylation via Pho85-Pho80 and is anchored to the 14-3-3 proteins in the cytoplasm when phosphorylated by TORC1-Sch9 [31,43]. Interestingly, a previous study reported that deletion of the 14-3-3 encoding genes *BMH1* or *BMH2* leads to heterogeneity in the expression of Pho4-regulated genes [114]. However, the nuclear localization of Pho4 triggered by loss of Sch9 activity is not sufficient to induce transcription of *PHO84* nor *PHO5*, both typical representatives of the PHO regulon. According to the *Saccharomyces* Genome Database Sch9 and Pho4 physically interact and based on a comprehensive mass spectrometry analysis of the rapamycin-sensitive phosphoproteome Pho4 has a perfect RRxS* consensus site for Sch9-mediated phosphorylation [62]. Hence, it is well possible that Sch9 directly targets Pho4 to control its activity. It is known that Pho4 is phosphorylated at different residues that control nuclear import or export and determine its transcriptional activity [67,113,115,116]. Export of Pho4 requires Msn5 [117] and, interestingly, as judged by our previously reported SGA analysis the combined deletion of *SCH9* and *MSN5* may result in a synthetic sick phenotype [11]. Alternatively, or in parallel, the lack of transcriptional induction by nuclear localized Pho4 in rapamycin-treated WT and *sch9Δ* cells can be due to an incompatible chromatin structure preventing Pho4 from having access to the promotors of *PHO84* and *PHO5*. It is known that the TORC1-Sch9 axis signals chromatin remodeling at many target genes via Ino80 and this appears to include at least *PHO5* [77,78].

For the expression of *GCN4*, our data confirm the previously reported results on its derepression during exponential growth upon loss of Sch9. Consistent with the priming by Pho85-Pho80 for subsequent TORC1-mediated activation of Sch9, a similar derepression is seen in the *pho80Δ* strain but in contrast to the *sch9Δ* strain, the derepression of *GCN4* in the *pho80Δ* is dependent on Pho4. Even so, the requirement of Pho4 was overruled by the additional deletion of *SCH9* in the *pho80Δ pho4Δ* strain, suggesting that Sch9 is the downstream target directly controlling *GCN4* expression and confining the role of Pho4 to fine-tuning via the PHO pathway. Given that Pho4 mediates a massive expression of the *PHO84* high-affinity phosphate transporter in the *pho80Δ* strain, we believe this fine-tuning can be explained by a model in which Pho84 phosphate uptake inhibits Pho81 in *pho80Δ* cells, leading to an active Pho85-Pcl7 kinase complex that in turn would lower the PI[3,5]P_2_ levels and thereby reduce the vacuolar recruitment and activation of Sch9 (Fig 8B). If true, then the reasons why loss of Pho4 rescues the synthetic lethality seen upon the combined deletion of *SCH9* and *PHO80* is to prevent the aberrant expression of Pho84, which otherwise results in failure to adjust PI[3,5]P_2_ levels in function of the TORC1-Sch9 output. It implies that phosphate uptake is normally strictly calibrated to the availability of other nutrient sources, including amino acids and nitrogen. That Gcn4 plays an important role here is underscored by the fact that also the stability of this short-lived transcription factor is under strict control of the phosphate sensing machinery as Pho85-Pcl5 triggers its nuclear degradation, while Pho81 and Pho85-Pcl7 are required to maintain its stability [29]. The calibration of phosphate uptake apparently also relates to the availability of fermentable carbon sources since Pho84 was shown to act as a transceptor that signals to PKA [118] and because enhanced PKA influences the downregulation and internalization of Pho84 from the plasma membrane [119]. The important role of Pho84 is reflected in another way as well. It is the main player connecting the retrograde response to replicative lifespan extension [120], the latter being equally dependent on PKA, Pho85-Pho80, TORC1, and Sch9 [121,122] and, obviously, also determined by telomere length [123]. Furthermore, it was shown that the additional deletion of *PHO4* partially restores the short-lived phenotype of the *pho85Δ* and *pho80Δ* strains [122], which is in line with our data.

### Concluding remark

This study started with the observation of synthetic lethality when the deletions of *SCH9* and *PHO85* or *PHO81* are combined [11]. Our data now provide a first glimpse of the crosstalk between these key players in nutrient signaling showing that this crosstalk is a complex but ingenious matter, dedicated to calibrating the responses triggered by a variety of nutritional signals through an interplay of processes at different levels. Our data demonstrate the importance of phosphatidylinositol metabolism to dictate the recruitment of Sch9 at vacuolar membrane, the consequence of Sch9 phosphorylation by Pho85 to prime for the subsequent phosphorylation and activation of Sch9 by TORC1, and the cooperation of Pho85 and TORC1-Sch9 signaling to control the nucleocytoplasmic translocation of Pho4 in a similar manner as previously described for Rim15 [31,43]. As such, it becomes evident that Sch9 functions as central integrator that allows to align different input signals and achieve accuracy in the responses. Given that Sch9 is also a substrate for the phytosphingosine-dependent kinases Pkh1, Pkh2, and Pkh3 [1,14,15] and the cellular energy sensor Snf1 [16–18], it will be interesting to elucidate how these inputs interfere with the Pho85-dependent processes described above.

## Materials and methods

### Yeast strains, plasmids, and growth conditions

The *Saccharomyces cerevisiae* strains used in this study for phenotypical analysis are listed in S1 Table. Deletion strains created for this study were generated using either polymerase chain reaction-based disruption cassettes, as previously described [124], or mating of haploid deletion strains of opposite mating types, followed by sporulation and tetrad analysis. Only deletion mutants with a BY4741 genotype (*his3Δ1 leu2Δ0 met15Δ0 ura3Δ0*) were used in subsequent experiments. The strains with the Sch9^S726A^ and Sch9^S726D^ point mutations were obtained by CRISPR/Cas9 [125]. For the creation of the Cas9 plasmid (pMC019; see S2 Table), the ‘SCH9-S726 Proto F’ and ‘SCH9-S726 Proto R’ primers were used (S3 Table). We co-transformed with both the plasmid and the corresponding single-stranded DNA donor sequences templates (S3 Table) for homology-directed repair. The point mutations in *SCH9* in plasmids pMC027, pMC028 and pMC029 were introduced by QuikChange kit (Agilent, Basel, Switzerland) by using plasmid pMC014 as template and the oligonucleotides ‘SCH9 mut T723A’, ‘SCH9 mut S726A’ and ‘SCH9 mut T723A-S726A’ (S3 Table). Yeast cells were transformed using the Gietz method [126]. Yeast cells were grown in YPD medium overnight and then diluted to 0.1 OD_600nm_ in the morning. Cells were grown to exponential phase, washed with sterile water, and then with 1 mL of 0.1 M LiAc. The corresponding μL of 10 OD_600nm_ of cells were used for transformation. The transformation mix contained 240 μL 50% PEG, 36 μL 1M LiAc, 2 μL of pRCC-K and 20 μL of donor sequence. Cells were incubated at 42°C for 40 min. After the transformation, the cells were re-suspended in YPD and grown at 30°C for 3 h and then plated onto YPD plates containing G418. To identify the clones containing the correct mutations in the *SCH9* region of interest was sequenced.

Many plasmids used in this study were a generous gift from other research groups (S2 Table). The centromere plasmids pRS413-Fab1 and pRS413-Fab1^VLA^ plasmids were subcloned from pRS416-*FAB1* and pRS416-*fab1-14* (kindly provided by L. Weisman, [55]). The plasmid for the expression of Fab1^VLA^-GFP under the control of its own promoter was created by PCR. Starting from a plasmid for the expression of Fab1-GFP, the backbone of the pRS416 plasmid, *FAB1* promoter, GFP, and *FAB1* terminator, were amplified by PCR. With a separate PCR, *FAB1-VLA* was amplified using pRS416-fab1-14 (provided by L. Weisman, [55]) as a template. The two PCR products were ligated with the Gibson Assembly Master Mix (New England Biolabs, Ipswich, USA) [127]. The ligation product was used for *E*. *coli* transformation and the plasmid was confirmed by sequencing. The construction of C-terminally FLAG-tagged (DYKDDDDK) versions of Sch9 (Sch9-FLAG) including Sch9^WT^, Sch9^T723A^, Sch9^S726A^, Sch9^T723A/S726A,^ Sch9^T737A^, Sch9^S758A^, and Sch9^S765A^ and cloning into the centromere plasmid pYCPlac33 has been described previously [18].

Yeast cells were grown in standard rich medium containing 2% bacto peptone, 1% yeast extract and 2% glucose (YPD) or in minimal medium containing 0.5% (NH_4_)_2_SO_4_, 1.9 g/l yeast nitrogen base without amino acids (Formedium, Norfolk, UK), supplemented with either synthetic selective drop-out mixtures (SD) or a complete synthetic mixture (SC) (Formedium, Norfolk, UK) as required, and 2% glucose. Solid medium contained an additional 1,5% agar. For phosphate starvation, the cells were grown to mid-log phase on SC medium and then transferred to yeast nitrogen base with ammonium sulphate and without phosphates (Formedium, Norfolk, UK) supplemented with 0.5% ammonium sulfate, the complete synthetic amino acid mixture and 4% glucose. For nitrogen starvation, the cells were transferred to yeast nitrogen base without amino acids and without ammonium sulfate (Formedium, Norfolk, UK) supplemented with 4% glucose. For carbon source starvation, the cells were transferred to SC medium without glucose.

### Rapamycin sensitivity analysis

Cells were grown to mid-log phase in either YPD, SD or SC medium, diluted to an optical density 600 nm (OD_600nm_) of 0.1. and serial dilutions (1:10) were spotted onto YPD, SD or SC plates with or without different concentrations of rapamycin as indicated and imaged after 3 to 5 days of growth at 30°C.

### Tetrad analysis

The diploids to assess genetic interaction of Sch9 with the Pho85-cyclins were generated by crossing either *sch9*::*HIS3* (BY4741, JW 01 306) or *sch9*::*LEU2* (BY4741, JW 01 307*)* with single cyclin deletion mutants form the Yeast Knock-Out Collection (YKO; EUROSCARF, BY4742). Similarly, the deletion strains of the genes identified as possible mutual targets of Pho85 and TORC1-Sch9 were obtained from the YKO Collection (EUROSCARF, BY4742), and crossed with either *pho85*::*KANMX4* (BY4741, JW 03 595) or *pho80*::*HIS3* (BY4741, JW 03 721). Sporulation was induced by spotting and incubating diploid cells on sporulation plates containing 1% potassium acetate, 0.1% KHCO_3_, pH 6.0 for 5–6 days at 25°C. Tetrads were treated with 0.02 mg/ml lyticase for 10 min at room temperature and were dissected on a YPD plate using a micromanipulator (Singer Instruments). After 3–5 days, the germinated spores were genotyped by plating them on the specific selective media and/or by PCR analysis. At least 6 tetrads were analyzed, and representative spores are shown in the pictures.

### Phos-tag and Western blot analysis

To analyze differential phosphorylation of Sch9 with Phos-tag SDS-PAGE, cells expressing either HA-Sch9 or Sch9-FLAG were grown on synthetic medium to mid-log phase. For the experiments with HA-tagged Sch9, cells were collected and washed with ice cold PBS and subsequently snap frozen in liquid nitrogen. A bead beating based lysis technique was used for protein extraction using a Triton-Deoxycholate buffer (50 mM HEPES pH7.4; 13.5 mM NaCl; 1% Triton X-100; 0.05% sodium deoxycholate), complemented with a protease and phosphatase inhibitor cocktail (Thermo Fisher Scientific, Merelbeke, Belgium). The cell lysates were cleared by a couple of subsequent centrifugation steps. Protein concentration was measured with the Bradford method (Bio-Rad, Temse, Belgium) and the samples were diluted to the same protein concentration in lysis buffer supplemented with Laemmli loading buffer. Samples were run on a 6,5% SDS-PAGE gel containing 25 μM Phos-tag (Fujifilm Wako Chemicals, Neuss, Germany). Full length HA-Sch9 was detected using an anti-HA-antibody (Roche, Merck, Hoeilaart, Belgium). For the experiments with FLAG-tagged Sch9, cells were heat-inactivated prior to collection and the preparation of protein extracts followed a protocol described previously [18]. Detection was done using an anti-FLAG antibody (Agilent, Basel, Switzerland). Both methods yielded comparable results.

For the analysis of Sch9 phosphorylation levels, cells expressing GFP-Sch9, GFP-FYVE-Sch9 or only endogenous Sch9 were grown to mid-log phase on synthetic medium. Cell lysate preparation was done as previously described, using bead beating in urea lysis buffer [61]. The phosphospecific anti-Sch9-P-Thr^737^ and anti-Sch9 antibodies [61,128], and the anti-GFP antibody (Roche, Merck, Hoeilaart, Belgium) were used to detect phosphorylated, endogenous Sch9, and GFP-Sch9 respectively after running the samples on an SDS-PAGE gel. Densitometry measurements were done with ImageJ to quantify the phosphorylation levels. The anti-GFP antibody was also used to determine the expression levels of the Fab1-GFP and Fab1^VLA^-GFP constructs as compared to the loading control Adh2 (anti-Adh2 antibody, Millipore, Merck, Hoeilaart, Belgium). For the detection of Atg13 and Lst4 phosphorylation levels, the strains were transformed with plasmids expressing the tagged constructs Atg13-HA_3_ or Lst4-V5, respectively. Sample preparations, detection using the anti-HA or anti-Lst4-P-Ser^523^ antibodies and quantifications were done as previously described [62,66].

### Protein purification

HA_2_-Pho85, HA_2_-Pho85^E53A^ (kinase-dead), and Pho80-GST were purified based on the description in [31]. The *pho85Δ* strain was transformed with plasmids pVW883, pVW884, and p946 (S2 Table). Cells were grown overnight in SD -Ura liquid medium. In the morning cells were diluted at 0.2 OD_600nm_ in 2 L SD -Ura. To induce Pho80-GST expression, cells were treated with 500 μM CuSO_4_ for 1 h, before harvesting the cells. Cells were collected by filtration, frozen in liquid nitrogen, and cryogenically disrupted by using a Precellys homogenizer in 10 ml of lysis buffer (50 mM Tris-HCl pH 7.5, 150 mM NaCl, 0.5 mM EDTA, 0.1% NP-40, 10% glycerol, 1 mM PMSF, 1 mM DTT, 400 mM Pefabloc, Roche complete protease inhibitor EDTA-free) in the presence of acid-washed glass beads. The cleared lysate was incubated for 2 h at 4°C with anti-HA magnetic beads (Fisher Scientific AG, Basel, Switzerland) for HA_2_-Pho85 and HA_2_-Pho85^E53A^ purifications and glutathione magnetic agarose beads (Fisher Scientific AG, Basel, Switzerland) for Pho80-GST purification. After 5 washes with lysis buffer, HA-beads coupled with Pho85 or Pho85^E53A^ were resuspended in 250 μL of elution buffer (50 mM Tris-HCl pH 7.5, 150 mM NaCl) and stored at 80°C after addition of 10% glycerol. GST-coupled beads with Pho80 were eluted at room temperature in 250 μL of elution buffer (50 mM Tris-HCl pH 7.5, 150 mM NaCl, 10 mM L-glutathione reduced) for 2h.

Yeast cells bearing the plasmids for Sch9^R650-I824^-TAP expression were grown overnight in SRaffinose-Ura supplemented with 0.01% sucrose. The day after, at 0.2 OD_600nm_, 2% final galactose was added to the cells for 6 h, to induce Sch9^R650-I824^-TAP expression. Cells were collected by filtration, frozen in liquid nitrogen, and cryogenically disrupted by using Precellys homogenizer in 10 mL of lysis buffer (50 mM Tris-HCl pH 7.5, 150 mM NaCl, 0.5 mM EDTA, 0.1% NP-40, 10% glycerol, 400 mM Pefabloc, Roche complete protease inhibitor EDTA-free). The cleared lysate was incubated with IgG-coupled Dynabeads M-270 (Thermo Fisher Scientific, Basel, Switzerland) for 2h at 4°C. After 5 washes with lysis buffer, Sch9^R650-I824^ was eluted in 150 μL TEV buffer (50mM Tris-HCl pH 7.5, 0.5mM EDTA,) with 2% TEV protease and stored at 80°C after the addition of 10% glycerol. Purified proteins were separated by SDS-PAGE, and stained with Sypro Ruby (Invitrogen, Thermo Fisher Scientific, Basel, Switzerland) to perform a quantification.

### Kinase assay

Kinase assays were performed with HA_2_-Pho85- and HA_2_- Pho85^E53A^ -bound beads, as described in [31]. The reaction was performed in kinase buffer (50 mM Tris-HCl pH 7.5, 20 mM MgCl_2_, 1 mM DTT). The reaction was carried out with 50 ng of kinase and Pho80 and 40 ng of the substrate. By adding the ATP mix (final concentration in reaction: 1mM ATP, 10 μCi γ-[32^P^]-ATP) the reaction was started and performed for 30 min at 30°C. By adding 2X SDS-PAGE sample buffer, the reaction was stopped. Samples were denatured at 65°C for 10 min, proteins were separated by SDS-PAGE, stained with Sypro Ruby (Invitrogen, Thermo Fisher Scientific, Basel, Switzerland) to assess loading, and analyzed using a phospho-imager (Typhoon FLA 9500; GE Healthcare, Opfikon, Switzerland), as described in [22].

### Fluorescence microscopy

Localization of Sch9 was determined in cells either expressing GFP-Sch9 from a plasmid [1] or genomically. The genomically tagged *pho85Δ*, *pho80Δ*, and *pho81Δ* strains were generated by crossing the *SCH9*::*GFP-SCH9* and *SCH9*::*GFP-FYVE-SCH9* strains (generously provided by A. Matsuura) with the respective deletion strains. Pho4 localization was monitored in cells containing the p*PHO4pr-PHO4-GFP* plasmid [129]. To assess vacuolar morphology, cells were stained with the lipid interacting dye FM4-64 (Invitrogen, Thermo Fisher Scientific, Merelbeke, Belgium) for 1 h. For all these assays, cells were grown to mid-log phase (OD_600nm_ 1–2) in glucose-containing synthetic medium. In case a glucose starvation condition was included, cells were washed twice and starved for 1 h on medium lacking glucose.

Most images were generated using either a Leica DMi8 S platform fluorescence microscope equipped with a Leica DFC9000 camera or a Leica DM 4000B fluorescence microscope equipped with a Leica DFC 300G camera (Leica Microsystems, Diegem, Belgium). A LUT Fire was applied using ImageJ to compare the intensities of the GFP signal. When indicated pictures were deconvoluted using the Huygens software (version 18.10; Scientific Volume Imaging B.V., Hilversum, The Netherlands). Confocal images of the Fab1-GFP and Fab1^VLA^-GFP constructs were captured with an inverted Spinning Disk Confocal Microscope (Nikon Ti-E inverted microscope, VisiScope CSU-W1, Amstelveen, The Netherlands) equipped with a PCO.edge 4.2 sCMOS camera and a 100x 1.3 NA oil immersion Nikon CFI series objective.

### RNA extraction, RT-PCR and Northern analysis

Northern blot analysis was performed as described previously [5]. In short, yeast cultures were grown overnight on YPD. Cultures were then diluted and allowed to grow till an OD_600nm_ of 1.5. Then control samples were taken (-30 and -15 min). Next, rapamycin was added to a final concentration of 200 nM and samples were taken after 15, 30, 60, and 120 min. RNA extraction and Northern blotting were performed as described previously [43]. The filters were hybridized with ^32^P-dCTP-labelled probes, generated with the High Prime kit (Roche, Merck, Hoeilaart, Belgium). Primers used for generation of the probes are listed in S3 Table. After washing, the filters were exposed to X-ray films (AGFA, Mortsel, Belgium).

For RT-PCR for *PHO5* expression analysis, 300 ng of the total RNA was retro-transcribed using the first-strand cDNA Synthesis kit (Nzytech, Lissabon, Portugal). NZYSpeedy qPCR Green Master Mix SYBR green Master Mix (Nzytech, Lisssabon, Portugal) was used to perform quantitative PCR in an Applied Biosystems 7500 fast qPCR system (Merck Life Sciences, Algés, Portugal). Data were analyzed with the Δ2CT method and normalized to the expression of *ACT1*, *PDA1* and *TDH2* genes in the same sample. The primer pairs used are listed in S3 Table.

### GFP-Sch9 quantification at isolated vacuoles

Vacuoles of GFP-Sch9 expressing WT, *pho85Δ*, and *fab1Δ* cells were isolated as described before [130], with the exception of some minor changes. Yeast cultures were grown in YPD to approximately OD_600nm_ 1. Cells were harvested, washed once, and resuspended in 0,03 M Tris-HCl pH 8,9 containing 10 mM DTT. After a 10 min incubation at 30°C, cells were incubated at 30°C in spheroplasting buffer (YP 0.2% glucose; 0.6 M sorbitol; 50 mM KP_i_; 0.1 mM pefabloc; 6U zymolyase/OD_600nm_ unit) for at least 30 min. The collected spheroplastes are resuspended in 15% ficoll buffer (15% ficoll; 10 mM PIPES/KOH pH 6.8; 0.2 M sorbitol; 0.1 mM pefabloc, 0.1 μg/ml leupeptin, 10 μg/ml o-phenantrolin, 0.5 μg/ml pepstatin A), to which 50 μl of 0.4 mg/ml diethylaminoethyl (DEAE) dextran was added per 100 OD_600nm_ units of cells. After 2 min incubation on ice, followed by 2 min at 30°C, the spheroplast suspension was transferred to a transparent SW41 tube (Beckman Coulter, Suarlée, Belgium). 8% ficoll buffer, 4% ficoll buffer and 0% ficoll buffer were pipetted carefully on top to create a discontinuous ficoll gradient. The samples were centrifuged for 90 min at 30’000 rpm in a SW41 rotor, at 4°C (Beckman Coulter, Suarlée, Belgium). After collecting the vacuolar fraction from the 0% - 4% ficoll interphase, vacuolar vesicles were further concentrated by diluting ½ in 10 mM PIPES/KOH pH 6.8 and centrifugation for 10 min at 5200g, 4°C. The purity of the isolated vacuolar vesicles was monitored by Western analysis, using Anti-Vph1 (Abcam, Cambridge, UK), anti-ATP6V1A (Abcam, Cambridge, UK), anti-Porin (Invitrogen, Thermo Fisher Scientific, Merelbeke, Belgium), anti-Pma1 (kindly provided by B. André), anti-Dpm1 (Invitrogen, Thermo Fisher Scientific, Merelbeke, Belgium). Total protein concentrations were measured with the Bradford method (Bio-Rad, Temse, Belgium). The obtained vacuolar vesicles were diluted to 0.1 μg/μl in 10 mM PIPES/KOH pH 6.8 and stained with 8 μM FM4-64 (Invitrogen, Thermo Fisher Scientific, Merelbeke, Belgium). GFP and FM4-64 signal intensity was measured with the Fluoroskan Ascent FL Microplate Fluorometer (Thermo Fisher Scientific, Merelbeke, Belgium), using a 485/518 filter pair and 530/645 filter pair respectively. The GFP ratio’s relative to FM4-64 or protein content in each sample was determined to serve as a measure of GFP-Sch9 abundance at the vacuolar membrane.

## Supporting information

S1 Fig Genetic interaction of SCH9 with different players of the Pho85 signaling pathway. Diploids were generated by crossing the haploid *SCH9* deletion strain with haploid strains carrying either a *PHO85*, *PHO81*, a single cyclin deletion or double cyclin deletion. Tetrad analysis only revealed a genetic interaction between *SCH9* and *PHO85*, *PHO81*, *PHO80*, *PCL6 PCL7* and *PHO80 PCL7* as indicated in red. The dissected spores were grown on YPD plates and pictures were taken after 3 to 5 days at 30°C.
(TIF)

S2 Fig Interaction between the TORC1- and Pho85-signaling pathways. (A, B) The WT strain or mutant strains lacking Pho85, Pho80, Pho81, a single cyclin (A) or two partially redundant cyclins (B) were grown exponentially on YPD, diluted to an OD_600nm_ of 0.1 and tenfold serial dilutions were spotted on YPD plates without or with 50 nM rapamycin. The strains were grown for 2 to 4 days at 30°C. (C) Immunoblot analysis of exponentially growing WT, *pho85Δ*, *pho80Δ*, and *pho81Δ* cells expressing GFP-Sch9^WT^ from an episomal plasmid in addition to endogenous Sch9. The Sch9-Thr^737^ phosphorylation level of GFP-Sch9 and endogenous Sch9 was quantified based on densitometry of the anti-P-Sch9^T737^ and anti-Sch9 signals, and normalized to WT cells. The data are represented as mean ± standard deviation. Paired two-tailed student’s T tests were used to calculate significances (*, P < 0.1; **, P < 0.01; ***, P < 0.001).
(TIF)

S3 Fig Phosphorylation of GFP-Sch9 in cells with enhanced Fab1 activity and GFP-Sch9 abundance at the vacuolar membrane. (A) Immunoblot analysis of exponentially growing WT cells expressing either Fab1 or Fab1^VLA^ from a centromere plasmid. The Sch9-Thr^737^ phosphorylation was quantified based on densitometry of the anti-P-Sch9^T737^ and anti-Sch9 signals, and normalized to WT cells transformed with an empty vector. (B) Microscopic analysis of Sch9 localization in the WT, *pho85Δ*, *pho80Δ*, and *pho81Δ* strains expressing the genomically tagged GFP-Sch9 or GFP-FYVE-Sch9 fusion protein. Strains were grown to mid-log phase on complete synthetic medium. The lipophilic dye FM4-64 was used to visualize the vacuolar membrane. Pictures were deconvoluted using the Huygens software (version 18.10). A LUT Fire was applied using ImageJ in order to show the levels of the GFP signal. (C) Western blot analysis to assess purity of the isolated vacuolar vesicles. Vacuoles were purified from spheroplasted cells using a density gradient centrifugation method as described in the materials and methods section. The high abundance of 2 typical vacuolar membrane proteins (Vma1 and Vph1) in the isolated vacuolar fraction in comparison to the whole cell protein extract (= Input) confirmed the strong enrichment of vacuolar proteins in this fraction. The presence of ER (Dpm1), mitochondrial (Por1) and plasma membrane (Pma1) markers, on the other hand, was very low in the isolated vacuolar fraction. (D) Fluorescence microscopic pictures of the purified vacuoles of GFP-Sch9^WT^-expressing cells, confirming the presence of GFP-Sch9 at the membranes of purified vacuoles. Staining with the lipophilic FM4-64 dye confirms the integrity of the isolated vacuolar vesicles. The intensity of the GFP-signal was quantified with a Fluoroskan plate reader as explained in the materials and methods section and expressed relative to the FM4-64 signal as well as the total protein content. The data are represented as mean ± standard deviation. Paired two-tailed student’s T tests were used to calculate significances (*, P < 0.1; **, P < 0.01; ***, P < 0.001).
(TIF)

S4 Fig Pho85-Pho80-mediated phosphorylation of Ser^726^ primes Sch9 for its subsequent activation by TORC1. (A) Phos-tag immunoblot analysis of protein extracts obtained from exponentially growing *sch9Δ* cells transformed with a centromere plasmid allowing for expressing C-terminally FLAG-tagged Sch9^T723A^, Sch9^S726A^, Sch9^T737A^, Sch9^S758A^, Sch9^S765A^, or Sch9^WT^ (WT) as indicated. Total protein extracts were resolved on phos-tag gels and were subsequently analyzed via immunoblotting with an anti-FLAG antibody. (B) Setup for the *in vitro* kinase assay to demonstrate phosphorylation of Sch9 by Pho85-Pho80. Various mixtures of purified HA-tagged Pho85 or the kinase dead (KD) Pho85^E53A^ mutant, GST-tagged Pho80, and a TAP-tag purified fragment corresponding to the C-terminus (CT) of Sch9 (Arg^650^ to Ile^824^) were used. The SYPRO Ruby staining and ^32^P autoradiograph are shown. (C) Microscopic analysis of WT (BY4741) cells expressing genomically tagged GFP-Sch9^WT^ or the mutant versions GFP-Sch9^S726A^ or GFP-Sch9^S726D^ showing the recruitment at the vacuolar membrane of wild-type Sch9 as well as both Sch9 variants. The strains were grown to mid-log phase on complete synthetic medium. The lipophilic dye FM4-64 was used to visualize the vacuolar membrane. (D) Survival profiles of cells expressing either SCH9^WT^, Sch9^S726A^ or Sch9^S726D^ when maintained on complete synthetic medium or starved for carbon (C), nitrogen (N) or phosphate (P).
(TIF)

S5 Fig The Pho85-cyclins Pcl6 and Pcl7 contribute the regulation of Sch9. (A) Immunoblot analysis of protein extracts from the WT, *pcl6Δ*, *pcl7Δ*, and *pcl6Δ pcl7Δ* strains exponentially growing on complete synthetic medium to assess changes in Sch9 phosphorylation. The Sch9-Thr^737^ phosphorylation levels were quantified based on the ratio of the anti-P-Sch9^T737^ and anti-Sch9 signals, and normalized to the ratio obtained for the WT cells. Paired two-tailed student’s T tests were used to calculate significances (*, P < 0.1; **, P < 0.01; ***, P < 0.001). (B) Immunoblot analysis of protein extracts of the WT strain and strains lacking non-essential Glc7-interacting proteins. The strains were grown to mid-log phase and were then treated with 200 nM rapamycin. Samples were taken before and after rapamycin treatment for 1 hour. The anti-Sch9 and anti-P-Sch9^T737^ antibodies were used for detection. The difference in mobility of the phosphorylated (P-Sch9) and non-phosphorylated (Sch9) isoforms as detected with the anti-Sch9 antibodies are indicated. (C) FM4-64 staining of the vacuolar membrane to show the difference in vacuolar size between WT cells and cells lacking Pho81, Pho85, or different combinations of Pho85 cyclins. Strains were grown to mid-log phase on complete synthetic medium containing 2% glucose. (D) Immunoblot analysis of the WT, *pho80Δ*, *pcl7Δ*, and *pho80Δ pcl7Δ* strains to compare the expression levels of the Fab1-GFP and Fab1^VLA^-GFP fusions when introduced on centromere plasmids as based on the ratio of the anti-GFP and anti-Adh2 signals. (E) Microscopic analysis of Fab1^VLA^-GFP localization in the WT, *pho80Δ*, *pcl7Δ*, and *pho80Δ pcl7Δ* strains. Strains were grown to mid-log phase on selective synthetic medium. The lipophilic dye FM4-64 was used to visualize the vacuolar membrane. The indents are magnifications showing that Fab1^VLA^ mainly localizes in foci at the periphery of small emerging vacuoles.
(TIF)

S6 Fig Analysis of downstream Pho85 effectors for the interplay with TORC1 signaling. (A) Diploids were generated by crossing the haploid *sch9Δ* strain with the *pho85Δ rim15Δ*, or the *pho85Δ crz1Δ* strain followed by genotype analysis of the dissected germinated spores. (B) Rapamycin sensitivity analysis of cells lacking Rim15 or Crz1 in a WT, *pho85Δ*, or *pho80Δ* background as determined by spot assays on YPD plates without or with 50 nM rapamycin. (C) Expression of *PHO5* as determined by RT-PCR in WT, *pho85Δ*, *pho80Δ*, and *sch9Δ* cells carrying an empty vector, or *sch9Δ* cells transformed with a centromere plasmid encoding Sch9^WT^ when grown to mid-logarithmic phase in SD-Ura medium before or after treatment with 200 nM rapamycin for 30 min. The data are represented as mean ± standard deviation. Paired two-tailed student’s T tests were used to calculate significances (*, P < 0.1; **, P < 0.01; ***, P < 0.001).
(TIF)

S1 Table *S*. *cerevisiae* strains used in this study. (DOCX)

S2 Table Plasmids used in this study. (DOCX)

S3 Table Oligonucleotides used in this study. (DOCX)

S4 Table Data statistics. (XLSX)
