# Supplementary figures and images for: The nutrient-responsive CDK Pho85 primes the Sch9 kinase for its activation by TORC1
Source: PLoS Genet. 2023 Feb 15;19(2):e1010641. doi: 10.1371/journal.pgen.1010641 (PMC9974134; doi:10.1371/journal.pgen.1010641)

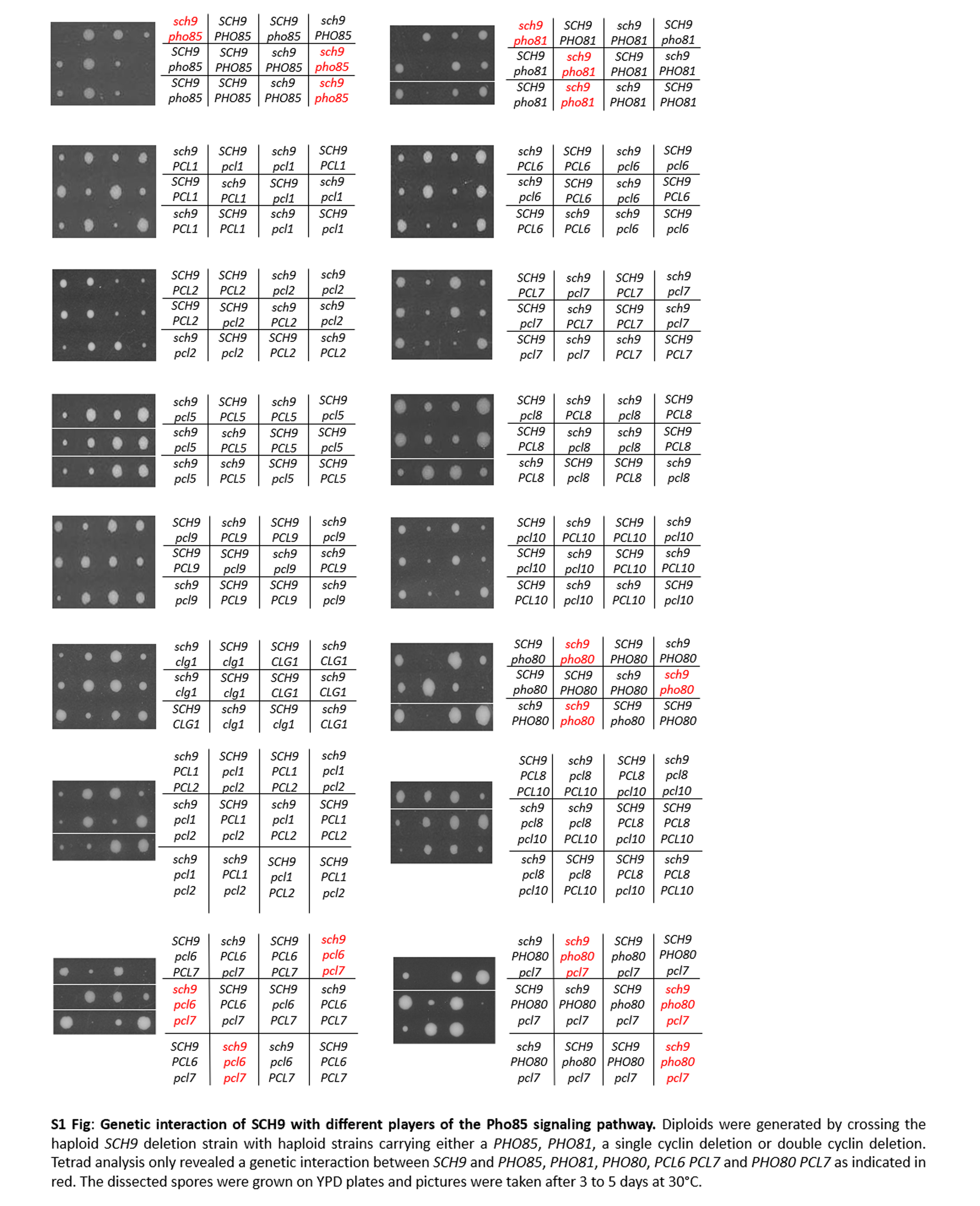

Supplement: S1 Fig — Diploids were generated by crossing the haploid SCH9 deletion strain with haploid strains carrying either a PHO85, PHO81, a single cyclin deletion or double cyclin deletion. Tetrad analysis only revealed a genetic interaction between SCH9 and PHO85, PHO81, PHO80, PCL6 PCL7 and PHO80 PCL7 as indicated in red. The dissected spores were grown on YPD plates and pictures were taken after 3 to 5 days at 30°C. (TIF) [file pgen.1010641.s001.tif]

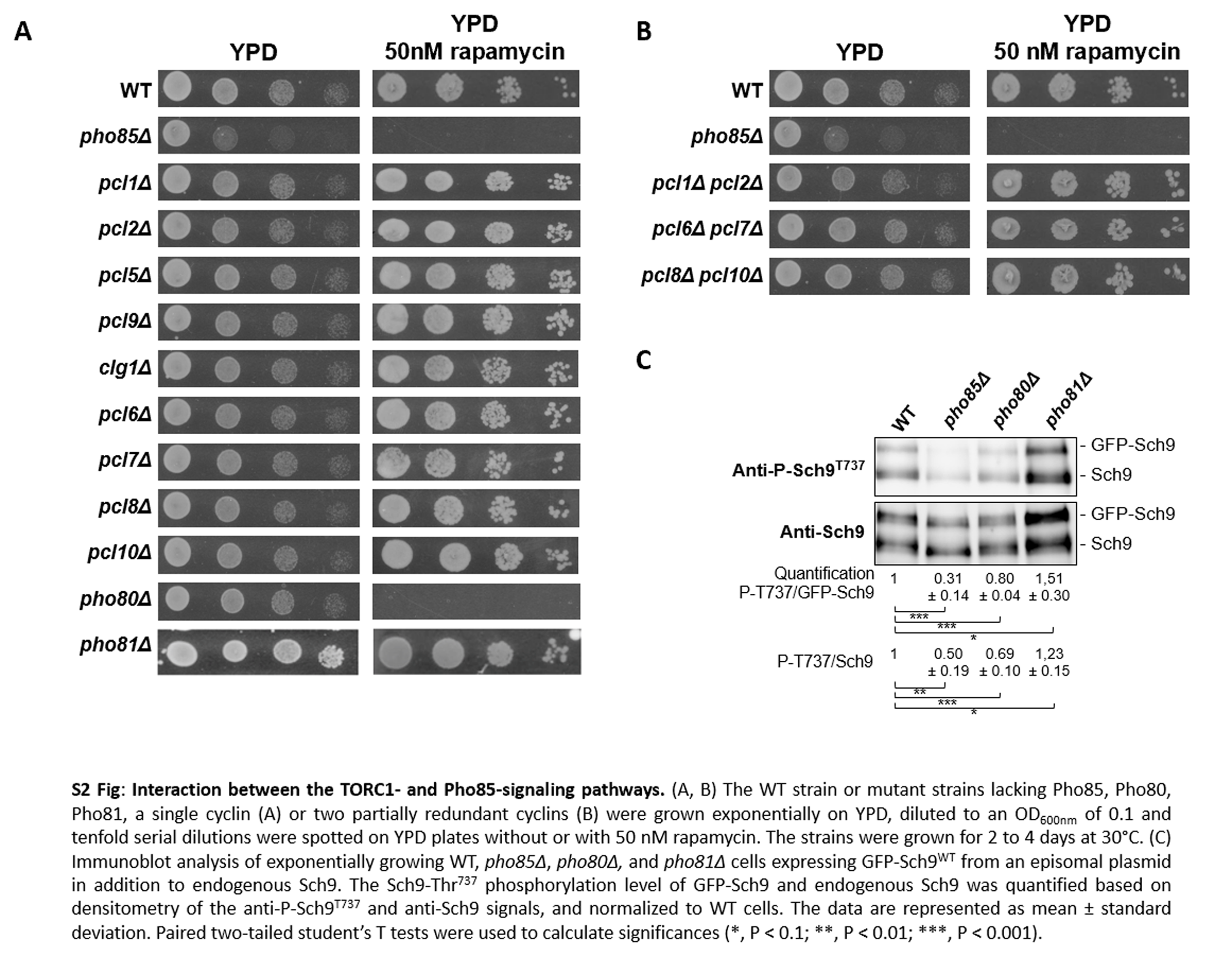

Supplement: S2 Fig — (A, B) The WT strain or mutant strains lacking Pho85, Pho80, Pho81, a single cyclin (A) or two partially redundant cyclins (B) were grown exponentially on YPD, diluted to an OD600nm of 0.1 and tenfold serial dilutions were spotted on YPD plates without or with 50 nM rapamycin. The strains were grown for 2 to 4 days at 30°C. (C) Immunoblot analysis of exponentially growing WT, pho85Δ, pho80Δ, and pho81Δ cells expressing GFP-Sch9WT from an episomal plasmid in addition to endogenous Sch9. The Sch9-Thr737 phosphorylation level of GFP-Sch9 and endogenous Sch9 was quantified based on densitometry of the anti-P-Sch9T737 and anti-Sch9 signals, and normalized to WT cells. The data are represented as mean ± standard deviation. Paired two-tailed student’s T tests were used to calculate significances (*, P < 0.1; **, P < 0.01; ***, P < 0.001). (TIF) [file pgen.1010641.s002.tif]

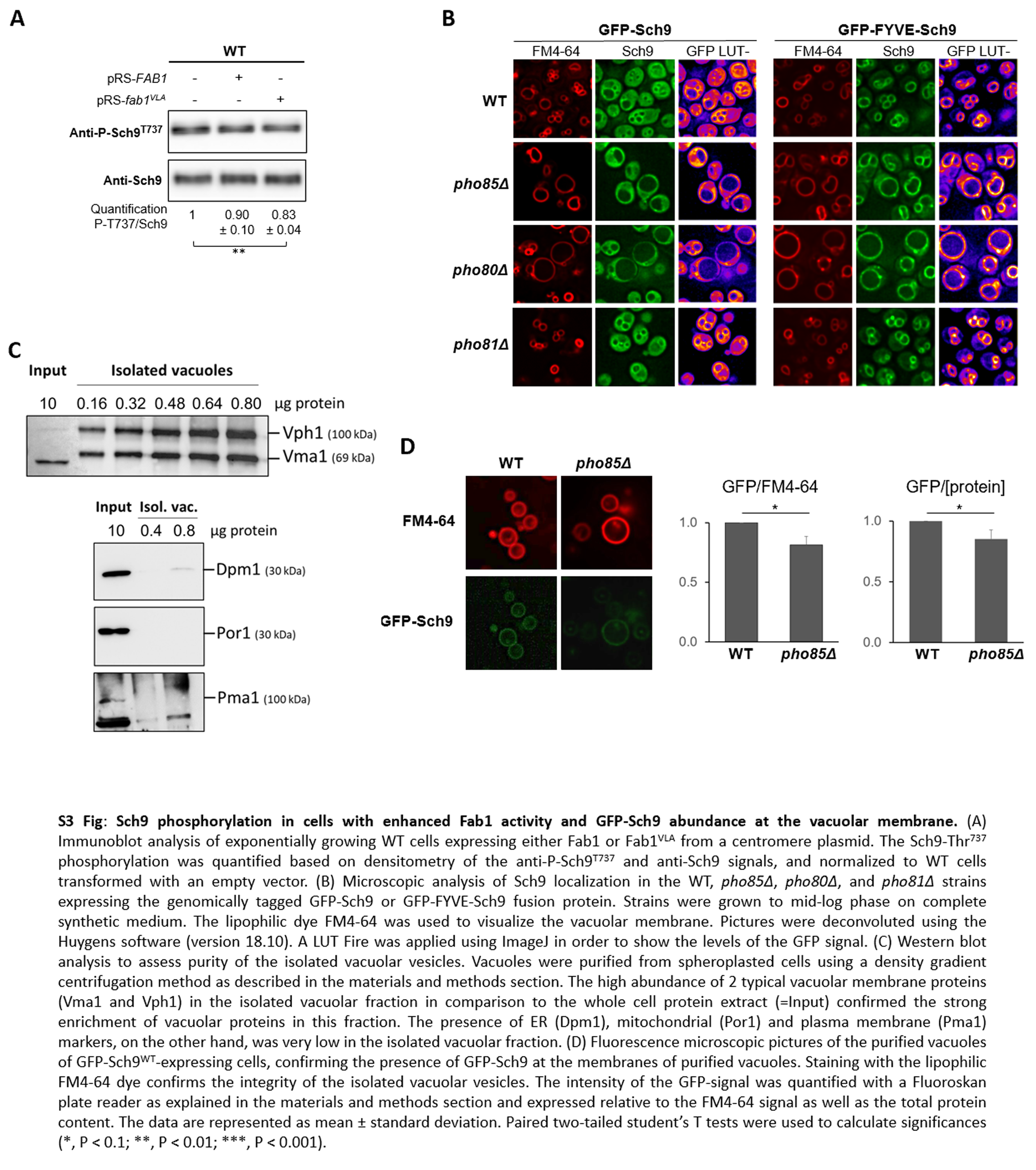

Supplement: S3 Fig — (A) Immunoblot analysis of exponentially growing WT cells expressing either Fab1 or Fab1VLA from a centromere plasmid. The Sch9-Thr737 phosphorylation was quantified based on densitometry of the anti-P-Sch9T737 and anti-Sch9 signals, and normalized to WT cells transformed with an empty vector. (B) Microscopic analysis of Sch9 localization in the WT, pho85Δ, pho80Δ, and pho81Δ strains expressing the genomically tagged GFP-Sch9 or GFP-FYVE-Sch9 fusion protein. Strains were grown to mid-log phase on complete synthetic medium. The lipophilic dye FM4-64 was used to visualize the vacuolar membrane. Pictures were deconvoluted using the Huygens software (version 18.10). A LUT Fire was applied using ImageJ in order to show the levels of the GFP signal. (C) Western blot analysis to assess purity of the isolated vacuolar vesicles. Vacuoles were purified from spheroplasted cells using a density gradient centrifugation method as described in the materials and methods section. The high abundance of 2 typical vacuolar membrane proteins (Vma1 and Vph1) in the isolated vacuolar fraction in comparison to the whole cell protein extract (= Input) confirmed the strong enrichment of vacuolar proteins in this fraction. The presence of ER (Dpm1), mitochondrial (Por1) and plasma membrane (Pma1) markers, on the other hand, was very low in the isolated vacuolar fraction. (D) Fluorescence microscopic pictures of the purified vacuoles of GFP-Sch9WT-expressing cells, confirming the presence of GFP-Sch9 at the membranes of purified vacuoles. Staining with the lipophilic FM4-64 dye confirms the integrity of the isolated vacuolar vesicles. The intensity of the GFP-signal was quantified with a Fluoroskan plate reader as explained in the materials and methods section and expressed relative to the FM4-64 signal as well as the total protein content. The data are represented as mean ± standard deviation. Paired two-tailed student’s T tests were used to calculate significances (*, P < 0.1; **, P < 0.01; [file pgen.1010641.s003.tif]

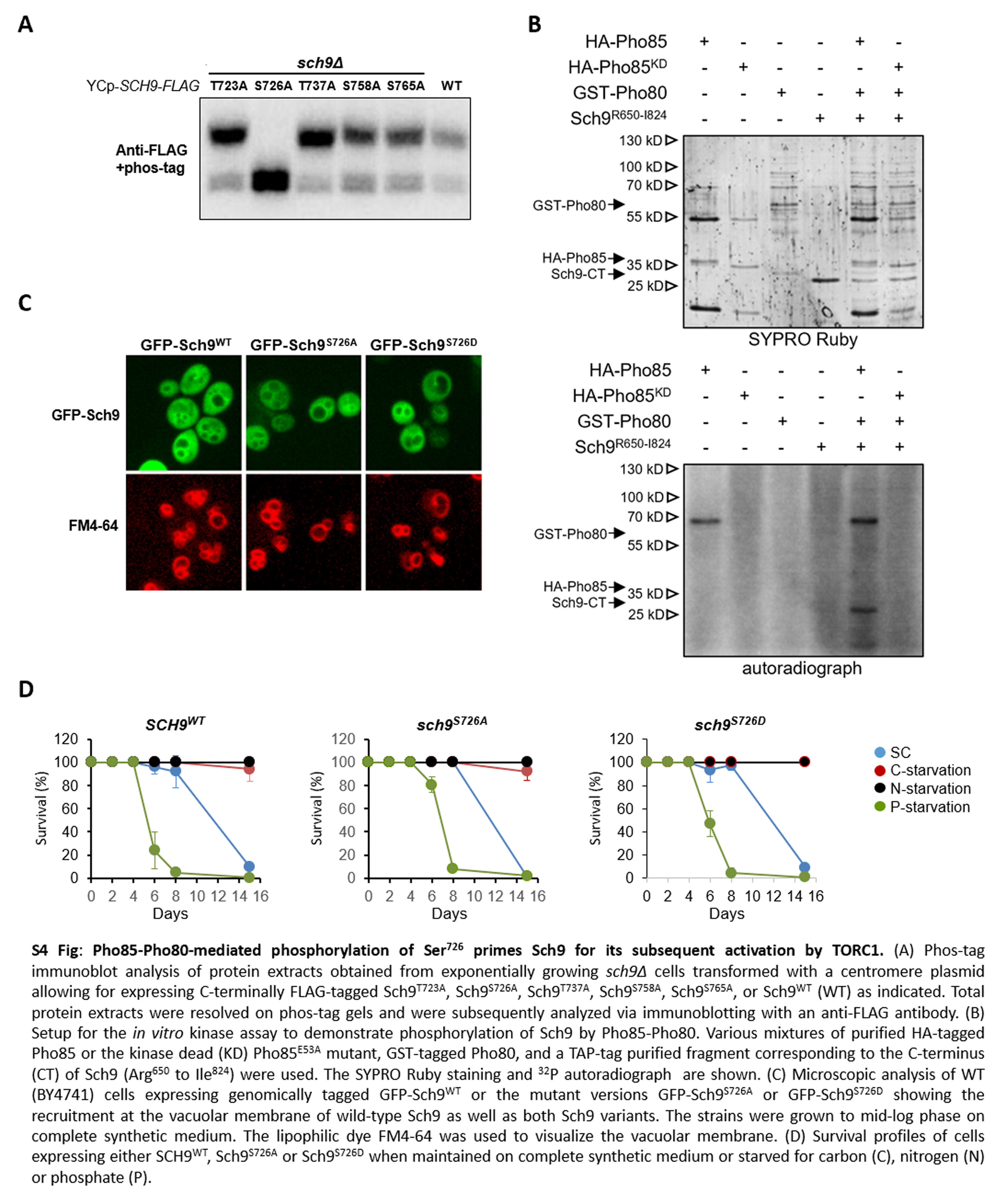

Supplement: S4 Fig — (A) Phos-tag immunoblot analysis of protein extracts obtained from exponentially growing sch9Δ cells transformed with a centromere plasmid allowing for expressing C-terminally FLAG-tagged Sch9T723A, Sch9S726A, Sch9T737A, Sch9S758A, Sch9S765A, or Sch9WT (WT) as indicated. Total protein extracts were resolved on phos-tag gels and were subsequently analyzed via immunoblotting with an anti-FLAG antibody. (B) Setup for the in vitro kinase assay to demonstrate phosphorylation of Sch9 by Pho85-Pho80. Various mixtures of purified HA-tagged Pho85 or the kinase dead (KD) Pho85E53A mutant, GST-tagged Pho80, and a TAP-tag purified fragment corresponding to the C-terminus (CT) of Sch9 (Arg650 to Ile824) were used. The SYPRO Ruby staining and 32P autoradiograph are shown. (C) Microscopic analysis of WT (BY4741) cells expressing genomically tagged GFP-Sch9WT or the mutant versions GFP-Sch9S726A or GFP-Sch9S726D showing the recruitment at the vacuolar membrane of wild-type Sch9 as well as both Sch9 variants. The strains were grown to mid-log phase on complete synthetic medium. The lipophilic dye FM4-64 was used to visualize the vacuolar membrane. (D) Survival profiles of cells expressing either SCH9WT, Sch9S726A or Sch9S726D when maintained on complete synthetic medium or starved for carbon (C), nitrogen (N) or phosphate (P). (TIF) [file pgen.1010641.s004.tif]

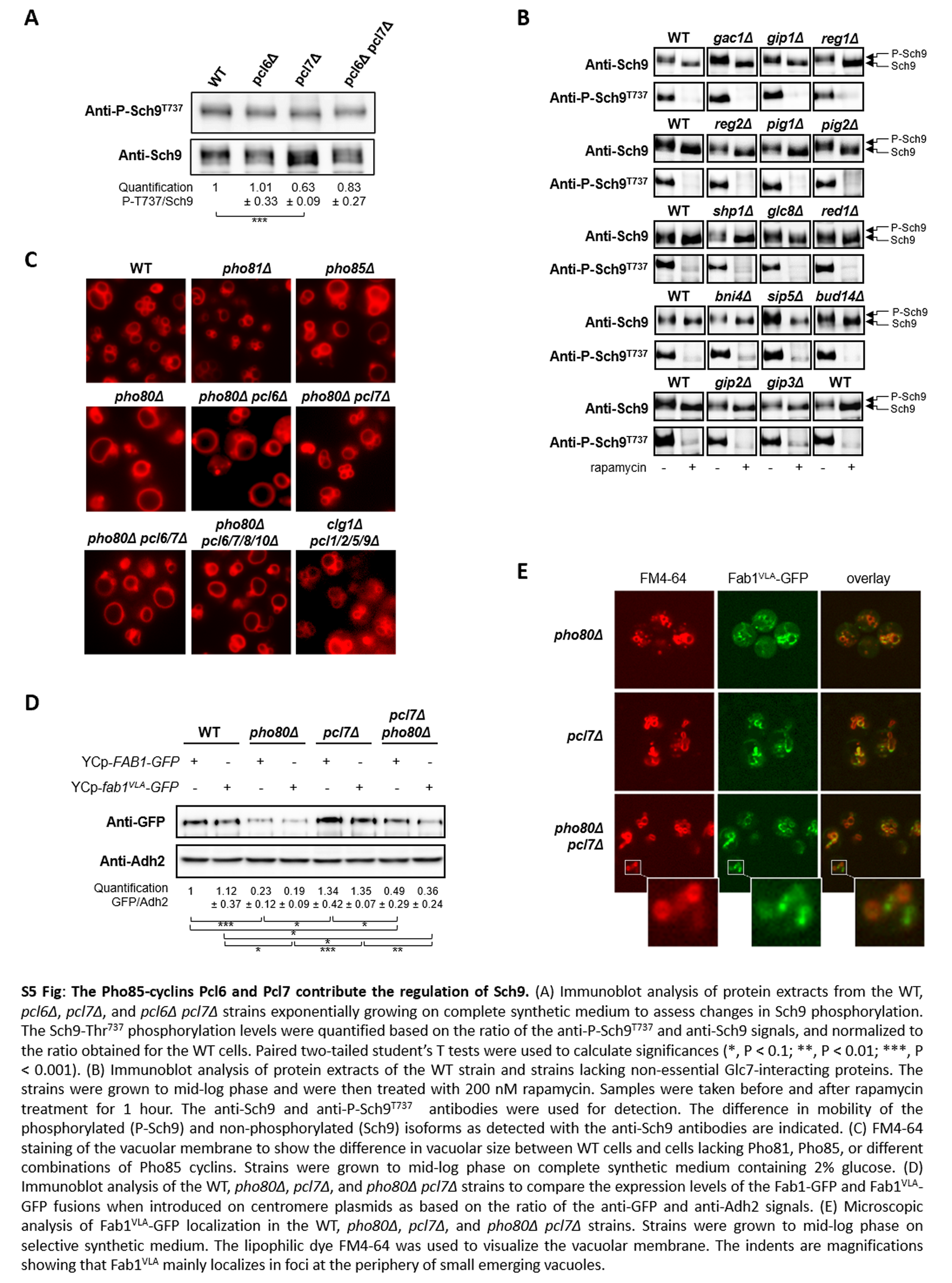

Supplement: S5 Fig — (A) Immunoblot analysis of protein extracts from the WT, pcl6Δ, pcl7Δ, and pcl6Δ pcl7Δ strains exponentially growing on complete synthetic medium to assess changes in Sch9 phosphorylation. The Sch9-Thr737 phosphorylation levels were quantified based on the ratio of the anti-P-Sch9T737 and anti-Sch9 signals, and normalized to the ratio obtained for the WT cells. Paired two-tailed student’s T tests were used to calculate significances (*, P < 0.1; **, P < 0.01; ***, P < 0.001). (B) Immunoblot analysis of protein extracts of the WT strain and strains lacking non-essential Glc7-interacting proteins. The strains were grown to mid-log phase and were then treated with 200 nM rapamycin. Samples were taken before and after rapamycin treatment for 1 hour. The anti-Sch9 and anti-P-Sch9T737 antibodies were used for detection. The difference in mobility of the phosphorylated (P-Sch9) and non-phosphorylated (Sch9) isoforms as detected with the anti-Sch9 antibodies are indicated. (C) FM4-64 staining of the vacuolar membrane to show the difference in vacuolar size between WT cells and cells lacking Pho81, Pho85, or different combinations of Pho85 cyclins. Strains were grown to mid-log phase on complete synthetic medium containing 2% glucose. (D) Immunoblot analysis of the WT, pho80Δ, pcl7Δ, and pho80Δ pcl7Δ strains to compare the expression levels of the Fab1-GFP and Fab1VLA-GFP fusions when introduced on centromere plasmids as based on the ratio of the anti-GFP and anti-Adh2 signals. (E) Microscopic analysis of Fab1VLA-GFP localization in the WT, pho80Δ, pcl7Δ, and pho80Δ pcl7Δ strains. Strains were grown to mid-log phase on selective synthetic medium. The lipophilic dye FM4-64 was used to visualize the vacuolar membrane. The indents are magnifications showing that Fab1VLA mainly localizes in foci at the periphery of small emerging vacuoles. (TIF) [file pgen.1010641.s005.tif]

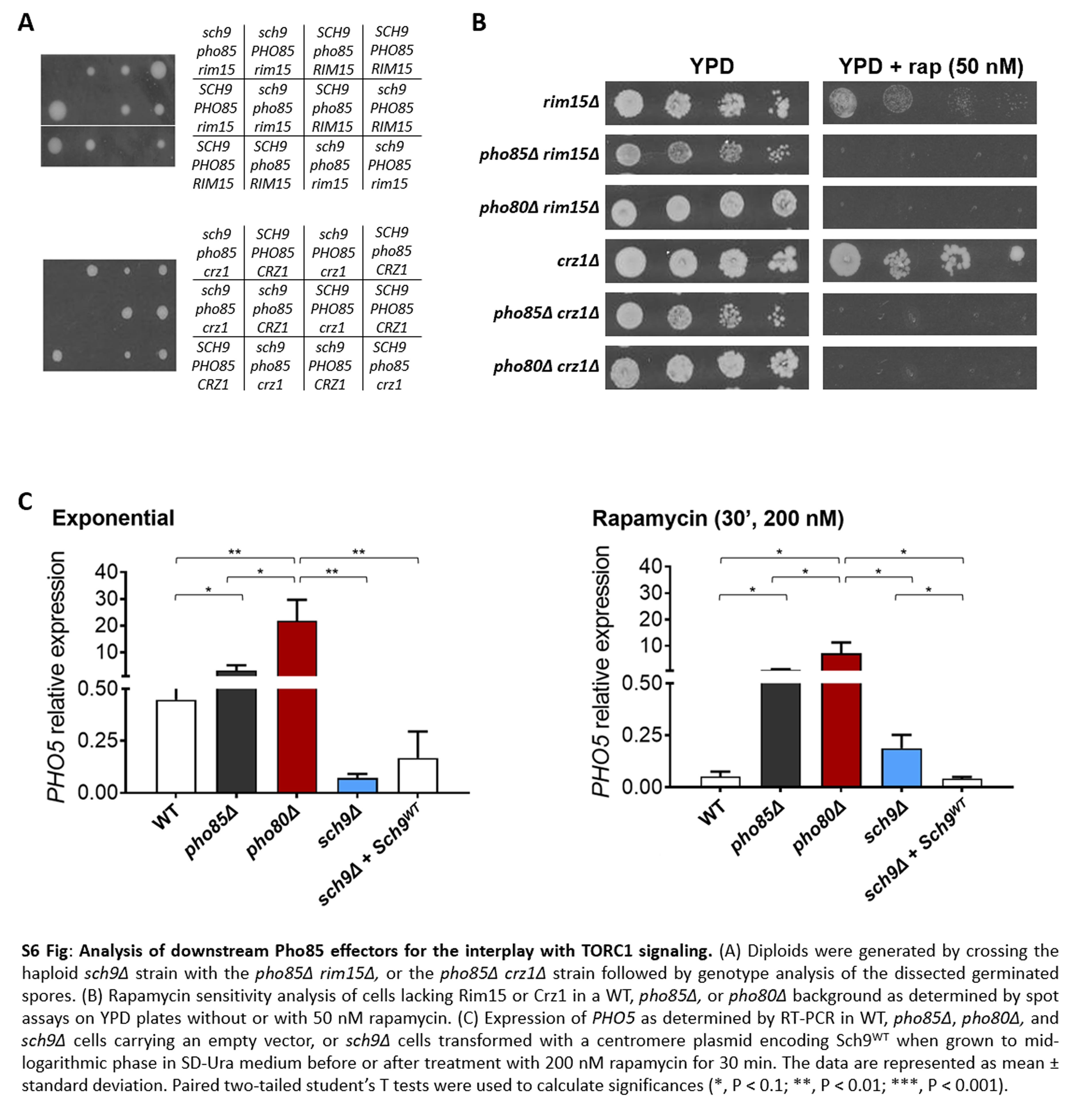

Supplement: S6 Fig — (A) Diploids were generated by crossing the haploid sch9Δ strain with the pho85Δ rim15Δ, or the pho85Δ crz1Δ strain followed by genotype analysis of the dissected germinated spores. (B) Rapamycin sensitivity analysis of cells lacking Rim15 or Crz1 in a WT, pho85Δ, or pho80Δ background as determined by spot assays on YPD plates without or with 50 nM rapamycin. (C) Expression of PHO5 as determined by RT-PCR in WT, pho85Δ, pho80Δ, and sch9Δ cells carrying an empty vector, or sch9Δ cells transformed with a centromere plasmid encoding Sch9WT when grown to mid-logarithmic phase in SD-Ura medium before or after treatment with 200 nM rapamycin for 30 min. The data are represented as mean ± standard deviation. Paired two-tailed student’s T tests were used to calculate significances (*, P < 0.1; **, P < 0.01; ***, P < 0.001). (TIF) [file pgen.1010641.s006.tif]
