# Supplementary material for: The nutrient-responsive CDK Pho85 primes the Sch9 kinase for its activation by TORC1
Source: PLoS Genet. 2023 Feb 15;19(2):e1010641. doi: 10.1371/journal.pgen.1010641 (PMC9974134; doi:10.1371/journal.pgen.1010641)
Supplement: S1 Table — (DOCX) [file pgen.1010641.s007.docx]

**S1 Table: *S. cerevisiae* strains used in this study**

| **Name** | **Genotype** | **Source** |
| --- | --- | --- |
| BY4741 (WT) | MATa *his3Δ1 leu2Δ0 met15Δ0 ura3Δ0* | [1] Openbiosystems [1] |
| JW 04 038 | BY4741 *sch9::NATMX4* | [2] |
| JW 01 306 | BY4741 *sch9::HIS3* | [3] |
| JW 01 307 | BY4741 *sch9::LEU2* | [3] |
| JW 03 595 | BY4741 *pho85::KANMX4* | YKO collection |
| JW 12 432 | BY4741 *pho81::KANMX4* | YKO collection |
| JW 10 644 | BY4741 *pho80::KANMX4* | YKO collection |
| JW 03 721 | BY4741 *pho80::HIS3* | [4] |
| JW 03 604 | BY4741 *pcl1::KANMX4* | YKO collection |
| JW 03 605 | BY4741 *pcl2::KANMX4* | YKO collection |
| JW 12 175 | BY4741 *clg1::KANMX4* | YKO collection |
| JW 03 607 | BY4741 *pcl5::KANMX4* | this study |
| JW 03 608 | BY4741 *pcl6::KANMX4* | YKO collection |
| JW 03 609 | BY4741 *pcl7::KANMX4* | this study |
| JW 03 610 | BY4741 *pcl8::KANMX4* | YKO collection |
| JW 03 611 | BY4741 *pcl9::KANMX4* | YKO collection |
| JW 03 612 | BY4741 *pcl10::KANMX4* | this study |
| JW 03 675 | BY4741 *pcl1::KANMX4 pcl2::KANMX4* | this study |
| JW 03 684 | BY4741 *pcl6::KANMX4 pcl7::KANMX4* | this study |
| JW 03 685 | BY4741 MATα *pcl8::KANMX4 pcl10::KANMX4* | this study |
| JW 01 893 | BY4741 *pho80::HIS3 pcl6::KANMX4* | this study |
| JW 01 894 | BY4741 *pho80::HIS3 pcl7::KANMX4* | this study |
| JW 03 727 | BY4741 *pho80::HIS3 pcl6::KANMX4 pcl7:: KANMX4* | this study |
| JW 03 732 | BY4741 *pcl6::KANMX4 pcl7::KANMX4 pcl8::KANMX4 pcl10::KANMX4 pho80::HIS3* | this study |
| JW 03 747 | BY4741 *pcl1::KANMX4 pcl2::KANMX4 pcl3::KANMX4 pcl9::Leu2 clg1::KANMX4* | this study |
| JW 03 591 | BY4741 *pho4::KANMX4* | YKO collection |
| JW 14 686 | BY4741 *rim15::KANMX4* | YKO collection |
| JW 13 780 | BY4741 *crz1::KANMX4* | YKO collection |
| JW 03 664 | BY4741 *pho85::KANMX4 pho4::KANMX4* | this study |
| JW 03 673 | BY4741 *pho85::KANMX4 rim15::KANMX4* | [4] |
| RG85C1 | BY4741 *pho85::KANMX4 crz1::KANMX4* | this study |
| JW 02 334 | BY4741 *pho80::HIS3* *pho4::KANMX4* | this study |
| JW 03 710 | BY4741 *pho80::HIS3* *rim15::KANMX4* | [4] |
| JW 02 430 | BY4741 *pho80::HIS3* *crz1::KANMX4* | this study |
| JW 02 338 | BY4741 *sch9*::*LEU2* *pho80*::*HIS3* *pho4*::*KANMX4* | this study |
| JW 03 594 | BY4741 *pho84::KANMX4* | YKO collection |
| yet36 | BY4741 *SCH9::GFP-SCH9* | [5] |
| JW 05 300 | yet36 *pho85:: KANMX4* | this study |
| JW 05 304 | yet36 *pho80::KANMX4* | this study |
| JW 05 296 | yet36 *pho81::KANMX4* | this study |
| yet234 | BY4741 *SCH9::GFP-FYVE-SCH9* | [5] |
| JW 05 298 | yet234 *pho85::KANMX4* | this study |
| JW 05 302 | yet234 *pho80::KANMX4* | this study |
| JW 05 294 | yet234 *pho81::KANMX4* | this study |
| MC131 | BY4741 *sch9-S726A* | this study |
| MC127 | BY4741 *sch9-S726D* | this study |
| MB32 | BY4741 *gtr1::KANMX6* | [6] |
| RKH526 | BY4741 *atg13::KanMX* | this study |
| MC171 | BY4741 *ATG13-HA_3_::KanMX* | this study |
| MC172 | BY4741 *ATG13-HA_3_::KanMX pho80∆::LEU2* | this study |
| MC173 | BY4741 *ATG13-HA_3_::KanMX pho85∆::LEU2* | this study |
| MP347-1A | BY4741 *lst4::KanMX* | [7] |
| MC174 | BY4741 *LST4-V5::KanMX* | this study |
| MC175 | BY4741 *LST4-V5::KanMX pho80∆::LEU2* | this study |
| MC176 | BY4741 *LST4-V5::KanMX pho85∆::LEU2* | this study |
| RKH395 | BY4741 *LEU2::GFP-TOR1* | [8] |
| CDV5410 | BY4741 *FAB1-GFP::HIS3* | Euroscarf |
| yRL649 | BY4741 *LEU2::GFP-TOR1* | this study |
| yRL650 | BY4741*pho85::KANMX4 LEU2::GFP-TOR1* | this study |
| yRL714 | BY4741 *pho80::HIS3 LEU2::GFP-TOR1* | this study |

**References**

1. Brachmann CB, Davies A, Cost GJ, Caputo E, Li J, Hieter P, et al. Designer deletion strains derived from Saccharomyces cerevisiae S288C: a useful set of strains and plasmids for PCR-mediated gene disruption and other applications. Yeast. 1998;14(2):115-32. Epub 1998/03/04. doi: 10.1002/(SICI)1097-0061(19980130)14:2<115::AID-YEA204>3.0.CO;2-2. PubMed PMID: 9483801.

2. Wilms T, Swinnen E, Eskes E, Dolz-Edo L, Uwineza A, Van Essche R, et al. The yeast protein kinase Sch9 adjusts V- ATPase assembly/ disassembly to control pH homeostasis and longevity in response to glucose availability. PLOS Genetics. 2017;13(6):e1006835. doi: 10.1371/journal.pgen.1006835.

3. Smets B, De Snijder P, Engelen K, Joossens E, Ghillebert R, Thevissen K, et al. Genome-wide expression analysis reveals TORC1-dependent and -independent functions of Sch9. FEMS Yeast Res. 2008;8(8):1276-88. Epub 2008/09/02. doi: 10.1111/j.1567-1364.2008.00432.x. PubMed PMID: 18759743.

4. Swinnen E, Rosseels J, Winderickx J. The minimum domain of Pho81 is not sufficient to control the Pho85-Rim15 effector branch involved in phosphate starvation-induced stress responses. Curr Genet. 2005;48(1):18-33. doi: 10.1007/s00294-005-0583-3. PubMed PMID: 15926040.

5. Takeda E, Jin N, Itakura E, Kira S, Kamada Y, Weisman LS, et al. Vacuole-mediated selective regulation of TORC1-Sch9 signaling following oxidative stress. Mol Biol Cell. 2018;29(4):510-22. doi: 10.1091/mbc.E17-09-0553. PubMed PMID: 29237820; PubMed Central PMCID: PMCPMC6014174.

6. Binda M, Peli-Gulli MP, Bonfils G, Panchaud N, Urban J, Sturgill TW, et al. The Vam6 GEF controls TORC1 by activating the EGO complex. Mol Cell. 2009;35(5):563-73. doi: 10.1016/j.molcel.2009.06.033. PubMed PMID: 19748353.

7. Peli-Gulli MP, Sardu A, Panchaud N, Raucci S, De Virgilio C. Amino Acids Stimulate TORC1 through Lst4-Lst7, a GTPase-Activating Protein Complex for the Rag Family GTPase Gtr2. Cell Rep. 2015;13(1):1-7. doi: 10.1016/j.celrep.2015.08.059. PubMed PMID: 26387955.

8. Hatakeyama R, Peli-Gulli MP, Hu Z, Jaquenoud M, Garcia Osuna GM, Sardu A, et al. Spatially Distinct Pools of TORC1 Balance Protein Homeostasis. Mol Cell. 2019;73(2):325-38 e8. doi: 10.1016/j.molcel.2018.10.040. PubMed PMID: 30527664.
