# Supplementary material for: The nutrient-responsive CDK Pho85 primes the Sch9 kinase for its activation by TORC1
Source: PLoS Genet. 2023 Feb 15;19(2):e1010641. doi: 10.1371/journal.pgen.1010641 (PMC9974134; doi:10.1371/journal.pgen.1010641)
Supplement: S2 Table — (DOCX) [file pgen.1010641.s008.docx]

**S2 Table: Plasmids used in this study**

| **Name** | **backbone** | **marker** | **type, insert** | **source** |
| --- | --- | --- | --- | --- |
| pRS416 | pRS416 | *URA3* | CEN/ARS |  |
| pJU793 | pRS416 | *URA3* | CEN/ARS, *pSCH9pr-GFP-HA-SCH9^WT^* | R. Loewith [1] |
| pJU829 | pRS416 | *URA3* | CEN/ARS, *pSCH9pr-GFP-HA-SCH9^5A^* | R. Loewith [1] |
| pJU677 | pRS416 | *URA3* | CEN/ARS, *pSCH9pr-6HA-SCH9^WT^* | R. Loewith [1] |
| pJU790 | pRS416 | *URA3* | CEN/ARS, *pSCH9pr-6HA-SCH9^5A^* | R. Loewith [1] |
| pJU675 | pRS416 | *URA3* | CEN/ARS, *pSCH9pr-SCH9^WT^* | R. Loewith [1] |
| pJU822 | pRS416 | *URA3* | CEN/ARS, *pSCH9pr-SCH9^5A^* | R. Loewith [1] |
| pJU841 | pRS416 | *URA3* | CEN/ARS, *pSCH9pr-SCH9^2D3E^* | R. Loewith [1] |
| p2809 | pRS413 | *HIS3* | CEN/ARS, *pSCH9pr-GFP-SCH9^WT^* | this study |
| p4048 | pRS413 | *HIS3* | CEN/ARS, *pSCH9pr-GFP-SCH9^S726A^* | this study |
| p4049 | pRS413 | *HIS3* | CEN/ARS, *pSCH9pr-GFP-SCH9 ^S726D^* | this study |
| pYCPlac33-Sch9-FLAG | pYCplac33 | *URA3* | CEN/ARS, *SCH9-FLAG* +/-300bp | [2] |
| pYCPlac33-Sch9-T723A-FLAG | pYCplac33 | *URA3* | CEN/ARS, *SCH9-T723A-FLAG* +/-300bp | [2] |
| pYCPlac33-Sch9-S726A-FLAG | pYCplac33 | *URA3* | CEN/ARS, *SCH9-S726A-FLAG* +/-300bp | [2] |
| pYCPlac33-Sch9-T737AFLAG | pYCplac33 | *URA3* | CEN/ARS, *SCH9-T737A-FLAG* +/-300bp | [2] |
| pYCPlac33-Sch9-S758A-FLAG | pYCplac33 | *URA3* | CEN/ARS, *SCH9-S758A-FLAG* +/-300bp | [2] |
| pYCPlac33-Sch9-S765A-FLAG | pYCplac33 | *URA3* | CEN/ARS, *SCH9-S765A-FLAG* +/-300bp | [2] |
| pYCPlac33-Sch9-T723A/S726A-FLAG | pYCplac33 | *URA3* | CEN/ARS, *SCH9-T723A/S726A-FLAG* +/-300bp | [2], this study |
| EB0347 | pRS316 | *URA3* | CEN/ARS, *pPHO4pr-PHO4-GFP* | E. O’Shea [3] |
| *FAB1* | pRS416 | *URA3* | CEN/ARS, *FAB1* | L. Weisman [4] |
| *fab1-14*/*fab1^VLA^* | pRS416 | *URA3* | CEN/ARS, *fab1^E1822V/F1833L/T2250A^* | L. Weisman [4] |
| FBp1117 | pRS413 | *HIS3* | CEN/ARS, *FAB1* | this study |
| FBp1118 | pRS413 | *HIS3* | CEN/ARS, *fab1^E1822V/F1833L/T2250A^* | this study |
| P4879 | pRS416 | *URA3* | CEN/ARS, *pFAB1pr-FAB1-GFP-FAB1ter* | [5] |
| pMC038 | pRS416 | *URA3* | CEN/ARS, *pFAB1pr-fab1^VLA^-GFP-FAB1ter* | this study |
| pRCC-K | pRS42K | *KanMX* | 2µ, *pROX3pr-Cas9, SNR52pr-gRNA-SUP4ter* | E. Boles [6] |
| pMC019 | pRS42K | *KanMx* | [pRCC-K], 2µ, *pROX3pr-Cas9, SCH9-near-Ser726* | this study |
| pVW883 | pCM186 | *URA3* | CEN/ARS, *ptet07pr-HA_2_-PHO85* | [7] |
| pVW884 | pCM186 | *URA3* | CEN/ARS, *ptet07pr- HA2-pho85-E53A* | [7] |
| p946 | pYEX 4T-1 | *URA3* | 2µ, *pCUP1-PHO80-GST* | [8] |
| pMC014 | pYEplac195 | *URA3* | 2µ, *pGAL1pr-Sch9-R650-I824-TAP-Adh1ter* | this study |
| pMC027 | pYEplac195 | *URA3* | 2µ, *Sch9-R650-(T723A)-I824- TAP* | this study |
| pMC028 | pYEplac195 | *URA3* | 2µ, *Sch9-R650-(S726A)-I824- TAP* | this study |
| pMC027 | pYEplac195 | *URA3* | 2µ, *Sch9-R650-(T723A/S726A)-I824- TAP* | this study |

**References**

1. Urban J, Soulard A, Huber A, Lippman S, Mukhopadhyay D, Deloche O, et al. Sch9 is a major target of TORC1 in Saccharomyces cerevisiae. Mol Cell. 2007;26(5):663-74. doi: 10.1016/j.molcel.2007.04.020. PubMed PMID: WOS:000247378000006.

2. Mudholkar K, Fitzke E, Prinz C, Mayer MP, Rospert S. The Hsp70 homolog Ssb affects ribosome biogenesis via the TORC1-Sch9 signaling pathway. Nat Commun. 2017;8(1):937. doi: 10.1038/s41467-017-00635-z. PubMed PMID: 29038496; PubMed Central PMCID: PMCPMC5643326.

3. Kaffman A, Rank NM, O'Shea EK. Phosphorylation regulates association of the transcription factor Pho4 with its import receptor Pse1/Kap121. Genes Dev. 1998;12(17):2673-83. doi: 10.1101/gad.12.17.2673. PubMed PMID: 9732266; PubMed Central PMCID: PMCPMC317126.

4. Duex JE, Tang F, Weisman LS. The Vac14p-Fig4p complex acts independently of Vac7p and couples PI3,5P2 synthesis and turnover. J Cell Biol. 2006;172(5):693-704. doi: 10.1083/jcb.200512105. PubMed PMID: 16492811; PubMed Central PMCID: PMCPMC2063702.

5. Chen Z, Malia PC, Hatakeyama R, Nicastro R, Hu Z, Peli-Gulli MP, et al. TORC1 Determines Fab1 Lipid Kinase Function at Signaling Endosomes and Vacuoles. Curr Biol. 2021. doi: 10.1016/j.cub.2020.10.026. PubMed PMID: 33157024.

6. Generoso WC, Gottardi M, Oreb M, Boles E. Simplified CRISPR-Cas genome editing for Saccharomyces cerevisiae. J Microbiol Methods. 2016;127:203-5. doi: 10.1016/j.mimet.2016.06.020. PubMed PMID: 27327211.

7. Wanke V, Pedruzzi I, Cameroni E, Dubouloz F, De Virgilio C. Regulation of G0 entry by the Pho80-Pho85 cyclin-CDK complex. EMBO J. 2005;24(24):4271-8. doi: 10.1038/sj.emboj.7600889. PubMed PMID: 16308562; PubMed Central PMCID: PMCPMC1356330.

8. Tan YS, Morcos PA, Cannon JF. Pho85 phosphorylates the Glc7 protein phosphatase regulator Glc8 in vivo. J Biol Chem. 2003;278(1):147-53. doi: 10.1074/jbc.M208058200. PubMed PMID: 12407105.
