# Supplementary material for: The nutrient-responsive CDK Pho85 primes the Sch9 kinase for its activation by TORC1
Source: PLoS Genet. 2023 Feb 15;19(2):e1010641. doi: 10.1371/journal.pgen.1010641 (PMC9974134; doi:10.1371/journal.pgen.1010641)
Supplement: S3 Table — (DOCX) [file pgen.1010641.s009.docx]

S3 Table: Oligonucleotides used in this study

| **Name** | **Orientation** | **Sequence** |
| --- | --- | --- |
| **CRISPR/Cas9** | | |
| *SCH9-S726* Proto F | Forward | GCTTGCATGGCTGGAGATAGgttttagagctagaaatagcaagttaaaataagg |
| *SCH9-S726* Proto R | Reversed | CTATCTCCAGCCATGCAAGCgatcatttatctttcactgcggag |
| *SCH9-S726A* Donor | Reversed | aacaaaggtgaaaccagcaaactttgcttgcatggctggagCtagcggggtagcagtcatcatcggctggtgcttgttca |
| *SCH9-S726D* Donor | Reversed | aacaaaggtgaaaccagcaaactttgcttgcatggctggaTCtagcggggtagcagtcatcatcggctggtgcttgttca |
| *SCH9 mut T723A* | Reversed | TTGCATGGCTGGAGATAGCGGGGCAGCAGTCATCATCGGCTGGTG |
| *SCH9 mut S726A* | Reversed | AAACTTTGCTTGCATGGCTGGAGCTAGCGGGGTAGCAGTCATCAT |
| *SCH9 mut T723A-S726A* | Reversed | TTTGCTTGCATGGCTGGAGCTAGCGGGGCAGCAGTCATCATCGGC |
| **Northern blot analysis** | | |
| *ACT1* F | Forward | GCTGCTTTGGTTATTGATAACG |
| *ACT1* R | Reversed | AACCACCAATCCAGACGGAG |
| *PHO84* F | Forward | TGGAGAGGTGCCATCATGGG |
| *PHO84* R | Reversed | CGACCTTACCAGATGCAGCA |
| *GCN4* F | Forward | ATGTCCGAATATCAGCCAAGTTT |
| *GCN4* R | Reversed | TCAGCGTTCGCCAACTAATTTC |
| **RT-PCR** | | |
| *PHO5* F | Forward | TCAACATCACCTTGCAGACTGT |
| *PHO5* R | Reversed | TCGTAGTCCCAAGCAGGACA |
| *ACT1* F2 | Forward | GATCATTGCTCCTCCAGAA |
| *ACT1* R2 | Reversed | ACTTGTGGTGAACGATAGAT |
| *PDA1* F | Forward | TGACGAACAAGTTGAATTAGC |
| *PDA1* R | Reversed | TCTTAGGGTTGGAGTTTCTG |
| *TDH2* F | Forward | CCGCTGAAGGTAAGTTGA |
| *TDH2* R | Reversed | CGAAGATGGAAGAGTTAGAGT |
